# Cryo-EM snapshots of mycobacterial arabinosyltransferase complex EmbB_2_-AcpM_2_

**DOI:** 10.1007/s13238-020-00726-6

**Published:** 2020-05-03

**Authors:** Lu Zhang, Yao Zhao, Ruogu Gao, Jun Li, Xiuna Yang, Yan Gao, Wei Zhao, Sudagar S. Gurcha, Natacha Veerapen, Sarah M. Batt, Kajelle Kaur Besra, Wenqing Xu, Lijun Bi, Xian’en Zhang, Luke W. Guddat, Haitao Yang, Quan Wang, Gurdyal S. Besra, Zihe Rao

**Affiliations:** 1 State Key Laboratory of Medicinal Chemical Biology, College of Life Sciences and College of Pharmacy, Nankai University, Tianjin, 300353 China; 2 Shanghai Institute for Advanced Immunochemical Studies and School of Life Science and Technology, ShanghaiTech University, Shanghai, 201210 China; 3 CAS Center for Excellence in Molecular Cell Science, Shanghai Institute of Biochemistry and Cell Biology, Chinese Academy of Sciences (CAS), Shanghai, 200031 China; 4 University of Chinese Academy of Sciences, Beijing, 100101 China; 5 National Laboratory of Biomacromolecules and Key Laboratory of RNA Biology, CAS Center for Excellence in Biomacromolecules, Institute of Biophysics, CAS, Beijing, 100101 China; 6 Laboratory of Structural Biology, Tsinghua University, Beijing, 100084 China; 7 School of Biosciences, Institute of Microbiology and Infection, University of Birmingham, Birmingham, B15 2TT UK; 8 School of Chemistry and Molecular Biosciences, The University of Queensland, Brisbane, QLD 4072 Australia

**Keywords:** *Mycobacterium tuberculosis*, EmbB, cryo-EM, ethambutol, cell wall synthesis, arabinoglacatan, arabinosyltransferase, acyl-carrier-protein, drug discovery

## Abstract

Inhibition of *Mycobacterium tuberculosis (Mtb)* cell wall assembly is an established strategy for anti-TB chemotherapy. Arabinosyltransferase EmbB, which catalyzes the transfer of arabinose from the donor decaprenyl-phosphate-arabinose (DPA) to its arabinosyl acceptor is an essential enzyme for *Mtb* cell wall synthesis. Analysis of drug resistance mutations suggests that EmbB is the main target of the front-line anti-TB drug, ethambutol. Herein, we report the cryo-EM structures of *Mycobacterium smegmatis* EmbB in its “resting state” and DPA-bound “active state”. EmbB is a fifteen-transmembrane-spanning protein, assembled as a dimer. Each protomer has an associated acyl-carrier-protein (AcpM) on their cytoplasmic surface. Conformational changes upon DPA binding indicate an asymmetric movement within the EmbB dimer during catalysis. Functional studies have identified critical residues in substrate recognition and catalysis, and demonstrated that ethambutol inhibits transferase activity of EmbB by competing with DPA. The structures represent the first step directed towards a rational approach for anti-TB drug discovery.

**Electronic supplementary material:**

The online version of this article (10.1007/s13238-020-00726-6) contains supplementary material, which is available to authorized users.

## Introduction

Tuberculosis (TB) caused by *Mycobacterium tuberculosis* (*Mtb*) is, worldwide, the leading cause of human fatalities due to any infectious disease (WHO, 2018). Of great concern is the emergence of multi-drug-resistant (MDR)-TB and extensively drug-resistant (XDR)-TB, which has further exacerbated the global burden of TB and at the same time continues to lead to a reduction in clinical recovery rates (WHO, 2018).

**Figure 1:**
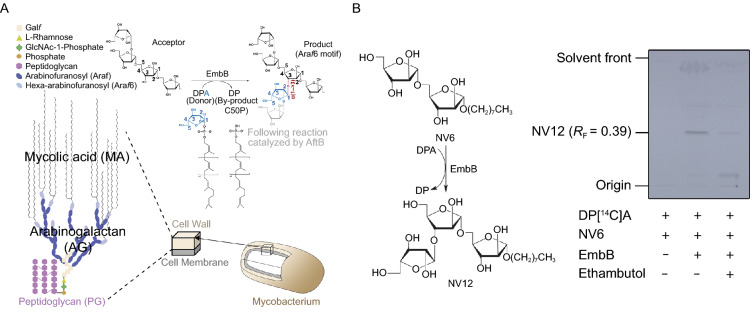
**Arabinosyltransferase activity of EmbB and inhibition by ethambutol**. (A) Schematic representation of the components and assembly of the mycobacterial membrane and cell wall. EmbB catalyzes the addition of an arabinose residue in an α(1→3) linkage from DPA resulting in the precursor for a subsequent extension by AftB, further resulting in the characteristic terminal branching hexamotif found in AG. (B) Arabinosyltransferase activity measured using di-arabinoside NV6. The [^14^C] labeled arabinose transferred from DP[^14^C]A to the product was confirmed by autoradiographic thin layer chromatography (TLC). See also Figures S1 and S9

Ethambutol (EMB) is one of the five front-line drugs used to treat TB and is particularly important in MDR-chemotherapy regime (Alliance, 2008). It exhibits its mode of action by inhibiting the biosynthesis of arabinogalactan (AG) (Takayama and Kilburn, 1989; Mikusova et al., 1995; Lee et al., 2005), a key component of the *Mtb* cell wall mycolyl-arabinogalactan-peptidoglycan (mAGP) complex (Jankute et al., 2015). However, the molecular basis for this inhibition has remained unresolved (Mikusova et al., 1995). Resistance to ethambutol has been shown to be caused by mutations within the *embCAB* operon (*embC*, *embA*, and *embB*) that encode membrane-associated arabinosyltransferases, amongst which EmbB has been identified as the primary target (Safi et al., 2008; Sun et al., 2018). Functional studies have shown that all the Emb proteins play key roles in cell wall synthesis. Specifically, EmbA and EmbB participate in arabinosylation of AG, which is linked via covalent attachment to the outer mycolic acids layer and the inner peptidoglycan layer, ultimately forming the cell wall core (Escuyer et al., 2001), thus acting as a natural barrier surrounding the cell membrane (Fig. 1A). On the other hand, EmbC is involved in the formation of lipoarabinomannan (LAM), a glycolipid which may modulate the host immune response during *Mtb* infection (Goude et al., 2008).

The *embB* gene has been shown to be essential for the survival of *Mtb* in culture (Sassetti et al., 2003), whereas a *Mycobacterium smegmatis* (*Msm*) *embB*-knockout strain was shown to be viable but possessed profound morphological alterations upon gene inactivation (Escuyer et al., 2001). Furthermore, EmbB has been shown to play a key role in forming the characteristic terminal hexarabinofuranosyl motif (Fig. 1A) of AG, which is the template for mycolylation (Escuyer et al., 2001; Jankute et al., 2015). Together with the other Emb proteins (EmbA and EmbC), EmbB belongs to the glycosyltransferase C (GT-C) superfamily (Berg et al., 2007; Lairson et al., 2008), whose structures comprises an N-terminal transmembrane domain and a C-terminal soluble domain located on the periplasmic side of the membrane (Berg et al., 2007). However, the Emb proteins show no overall sequence similarity to any other GT-C members or any other proteins beyond mycobacteria or related genera (Berg et al., 2005). The lipid donor utilized by EmbB is decaprenyl-phosphate-arabinose (DPA), which is the only proven arabinose donor for mycobacterial species (Lee et al., 1997). In a very recent study three dimensional structures of *Mtb* and *Msm* EmbA-EmbB heterodimer complexes and *Msm* EmbC_2_ homodimer complex were determined (Zhang et al., 2020). Nevertheless, *embA* knockout *Msm* (斜体) strain can survive, indicating EmbB protein can work alone in cell (Escuyer et al., 2001). However, other fashions of the Emb-containing assembly, *i.e.* (斜体), EmbB as an individual protein has not been reported.

Here, we have characterized EmbB in terms of its structure, catalytic mechanism and its inhibition by ethambutol. We present the cryo-EM structures of a full-length *Msm* EmbB in two distinct conformations, which we refer to as the “resting” and donor-bound “active” states at 3.6 Å and 3.5 Å resolution, respectively. EmbB is observed as a dimer along with an acyl-carrier-protein (AcpM) associated with each protomer, thus forming a heterotetrameric EmbB_2_-AcpM_2_ complex. We show that ethambutol inhibits the enzymatic activity of the EmbB_2_-AcpM_2_ complex and structurally identify the site that is most susceptible to ethambutol resistance based on isolates from clinical studies.

## Results and discussion

### Enzyme purification, characterization and structure determination

To gain insights into the structure and function of EmbB, we screened several mycobacterial orthologues to assess protein yield and purity. From these studies, we identified *Msm* EmbB (MSMEG_6389) as the ideal candidate for investigation. *Msm* EmbB, whose sequence is 69.6% identical to *Mtb* EmbB (Rv_3795) was cloned into the pMV261 vector containing a 10× His tag fusion at its C-terminus. *Msm* was then used as its host for overexpression (Snapper et al., 1990). Detergent purified followed by amphipol exchanged EmbB protein (Fig. S1A–C) was then subjected to cryo-EM analysis.

The natural acceptor of EmbB remains to be defined, but cell-free arabinosyltransferase activity could be measured using a diarabinoside, NV6, as an acceptor analog (Fig. 1B). An EmbB arabinosyltransferase assay was used to determine the transfer of [^14^C]-arabinose from DP[^14^C]A to NV6. The resulting product, NV12, was identified by autoradiographic thin-layer chromatography (TLC) (Fig. 1B). NV12 had a similar retardation factor to a synthetic tri-arabinoside suggesting the transfer of a single [^14^C]-arabinose unit from DP[^14^C]A, a feature common to these acceptor analogs (Lee et al., 1997). Not surprisingly, EmbB arabinosyltransferase activity is inhibited by ethambutol (Fig. 1B). Given NV6 allows three potential glycosylation sites at 2-OH, 3-OH and 5-OH on the terminal non-reducing arabinose, we sought to use a chemical biology approach to further characterize the [^14^C]-arabinose containing NV12 product. The related NV13 acceptor, where the 3-OH position of the terminal arabinose unit of NV6 is blocked by an azide group was used in subsequent cell-free experiments for purified EmbB, EmbC, and the abundant AftB activity from *Msm* membranes (Lee et al., 1997), to determine the resulting new glycosidic linkage in NV12 catalyzed by EmbB, which is presumably an α(1→3)-linkage based on previous studies (Escuyer et al., 2001). The azide group in NV13 prevented glycosylation by EmbB but allowed purified EmbC to catalyze an ethambutol-sensitive α(1→5)-linkage (NV15), and an ethambutol-resistant AftB β(1→2)-linkage (NV14) (Fig. S1E). In addition, 2D heteronuclear single quantum correlation (HSQC) NMR experiments using purified AG from wild type *Msm* and the *Msm embB* knockout strain were consistent with the above cell-free arabinosyltransferase data and showed that the cell wall from the knockout strain lacked the terminal linkage of arabinose unit by virtue of the absence of the characteristic 2-α-Ara*f*-3 NMR signal (Fig. S1F).

Purified, amphipol exchanged EmbB appeared as homogeneous and dispersed particles in negative staining EM and when embedded in vitreous ice (Fig. S2A). 2D class averages revealed a dimeric assembly of EmbB (Fig. S2B) with two attachments on the cytoplasmic side, subsequently identified by silver staining (Fig. S1C) and mass-spectrometry (Fig. S1D) as the endogenous acyl-carrier-protein AcpM (*MSMEG*_4326). Two major classes generated by 3D classification were selected and subjected to individual refinements, ultimately yielding two different reconstructions at 3.5 Å and 3.6 Å overall resolution (Fig. S2C-F). The quality of both maps allowed us to build, *de novo*, two near-atomic models of EmbB that include most of the residues (Tables S1 and S2), while AcpM could be docked and refined using a homologous structure (PDB: 1KLP (Wong et al., 2002)). Additional features in the cryo-EM map could be accounted for by the donor substrate, DPA, whose presence was confirmed by mass spectrometry (Fig. S1G). This feature shows DPA is bound in its expected binding cavity in the asymmetric EmbB dimer, and the potential by-product, decaprenyl phosphate (DP) bound to regions that are likely in the transmembrane domain of EmbB.

### Two distinct states of EmbB_2_-AcpM_2_ complex

**Figure 2:**
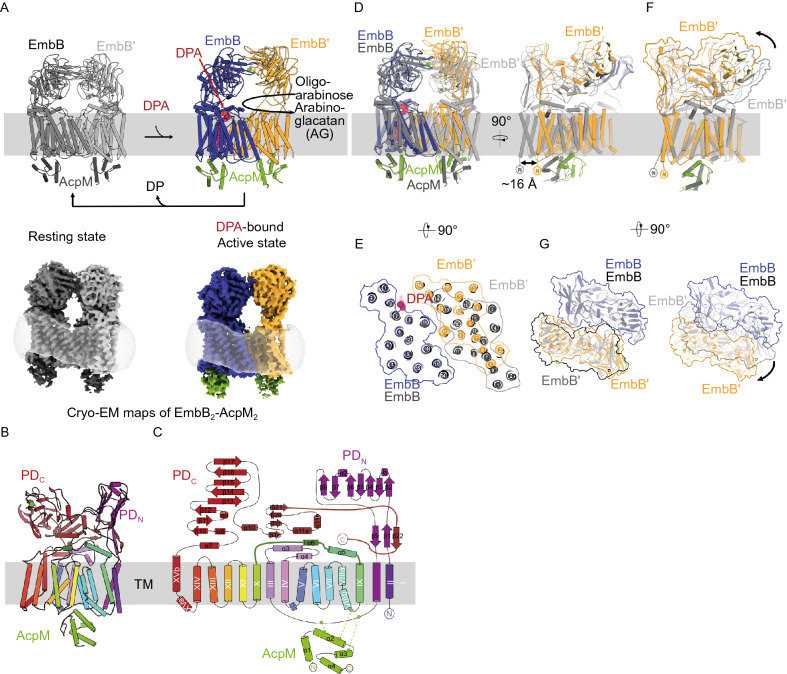
**Architecture and asymmetry in the EmbB**_**2**_**-AcpM**_**2**_**complex**. (A) Top row: Cartoon representation of the “resting” state (left) and “donor-bound” active state (right). DPA is drawn as red spheres. Bottom row: Cryo-EM maps of the two states, DPA is buried inside and is not shown in the map (bottom right). (B) Structure of an EmbB protomer viewed from the dimer interface, with a spectral coloring from purple (N terminus) to red (C terminus), the mixed α/β subdomain of PD_C_ has colored red and jellyroll fold subdomain in light brown. (C) Topology diagram for an EmbB protomer using the same color scheme as (B). (D) Superposition of the “resting” and “donor-bound” states. Left and right images have alternate views and (E) is from the top showing the transmembrane helices, and a translation of ~16 Å along with the dimer interface and in the plane of the membrane. (F) Superposition of the membrane portions of EmbB for the two unbound promoters shows the conformation change upon activation. (G) View from the periplasm, showing the movement of the periplasmic domain in black (resting state), grey (expected clash position) and yellow (active state). See also Figs. S2, S3 and S4 and Table S1, S2

The EmbB_2_-AcpM_2_ complex is captured in two states, one with C2-symmetry and the other that is asymmetric (Fig. 2A). In both reconstructions, each EmbB protomer is associated with an AcpM on its cytoplasmic side (Fig. 2A), resulting in a heterotetrameric assembly. Given their differences in composition and conformation, the two states are referred to as the asymmetric “donor-bound” active state and the symmetric “resting” state (Fig. 2A). The asymmetry refers not only to the asymmetric binding of DPA, but also to the asymmetry of the enzyme dimer assembly (see below). The EmbB protomers in the two states share common structural features which include two periplasmic domains (PDs) and 15 transmembrane helices (TMH) arranged into an approximately crescent-shaped bundle (Fig. 2B–E). The PDs comprise a PD_N_ located between TMH1 and TMH2 and a PD_C_ at the C-terminus (Fig. 2B and 2C). PD_C_ features a jelly-roll-fold subdomain coordinated with a Ca^2+^ ion, similar to the previously reported crystal structure of the C-terminal soluble domain of EmbC from *Mtb* (Alderwick et al., 2011), and a mixed α/β fold subdomain, whose interactions with both the TM domain and PD_N_ are observed here in the intact EmbB structure (Figs. 2C, S3B and S3C). PD_N_ also adopts a jelly-roll-fold and interacts with the C-terminal tail of PD_C_ by forming a three-stranded β-sheet (Figs. 2B, 2C and S3C), which may help to stabilize the entire PD. Not surprisingly, this overall folding represented by EmbB_2_-AcpM_2_ complex agrees well with that of the previously reported Emb proteins (Zhang et al., 2020).

The two EmbB_2_-AcpM_2_ complexes show significant differences in their subunit organizations (Fig. 2A, and 2D–G). Most significantly, the two EmbB protomers in the DPA bound complex are asymmetrically associated. Relative to the DPA-bound protomer, the other EmbB protomer is translated by ~16 Å along the dimer interface, and in the plane of the membrane (Fig. 2D and Movie S1). This change in subunit organization dramatically alters the dimer interface, in which, for instance, the distance between the pair of TMH11 at the interface has halved, resulting in a more compact active site (Fig. 2E). While this asymmetric movement is induced, the expected steric clashes in the periplasmic domains of the two EmbB protomers are resolved by rearrangements of the PLs and PDs within the unbound protomer (Fig. 2F, 2G, and Movie S1).

### DPA binding in the active complex

**Figure 3:**
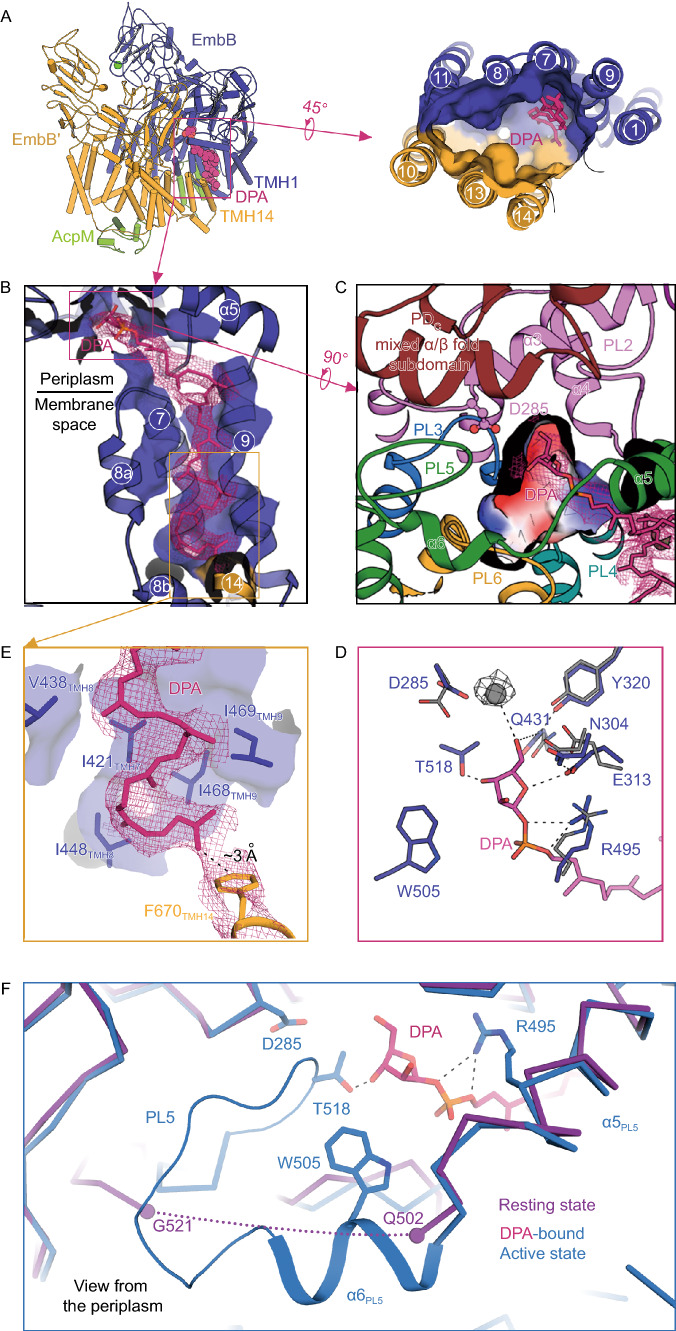
**DPA binding cavity in EmbB**. (A) Left: Cartoon representation of the EmbB_2_-AcpM_2_ complex in the “donor-bound” active state. Right: Cavity formed in the membrane space surrounded by TMH1, TMH7-9, and TMH11 from the DPA-bound EmbB protomer and the TMH10, TMH13, and TMH14 from the other protomer. DPA is in pink. (B) The complete DPA in the groove, with surrounding TMHs from both protomers. Cryo-EM map is shown in colored mesh. (C) The DPA-bound cavity viewed just above the membrane surface is bordered by PL2 which contains the catalytically relevant D(285)D(286)X motif, PL5 which is organized as two short α-helices (α5 and α6), and the mixed α/β subdomain of PD_C_. DPA is shown in pink. (D) Zoom-in view of the catalytic cavity, showing side-chains of residues interacting with the DPA head-group and a putative metal ion. Interactions are indicated by dashed lines. (E) The hydrophobic decaprenyl tail of DPA in the groove of the dimer interface, making several hydrophobic interactions, and a C-H···π-interaction with F670 from the empty protomer. (F) DPA binding cavity viewed from the periplasm. EmbB protomers from the “resting” state (purple) and “donor-bound” active state (blue) are superimposed. The dotted line from Q502 to G521 indicates a disordered loop in the “resting” state, which is stabilized in the active state through interactions with DPA. See also Figs. S1G, S5 and S8

In the “active” complex, a semi-confined gulf is formed in the membrane space that is surrounded by TMH1, TMH7-9 and TMH11 from the DPA-bound EmbB protomer and TMH10, TMH13, and TMH14 from the other protomer (Figs. 2E, 3A and 3B). Once the nearby side-chains were assigned (Fig. S4), the cryo-EM map shows a long tadpole-shaped signal in the hydrophobic groove formed by TMH7-9, heading to the presumed active site which is expected to be in the periplasmic space (Fig. 3B and 3C). Given that endogenous DPA was identified during purification (Fig. S1G), it was reasonable to build a DPA into this region (Fig. 3A and 3B). The whole of the DPA fits into this part of the map with the arabinose moiety and phosphate group fitting into a charged cavity just above the membrane surface (Fig. 3C and 3D). This cavity is bordered by PL2 which contains the catalytically relevant D(285)D(286)x motif (Berg et al., 2005); PL5 which is organized as two short α-helices (α5 and α6) and a following loop region; and the mixed α/β subdomain of PD_C_ (Fig. 3C). As a result of DPA binding, several residues around DPA are repositioned (Fig. 3D). For instance, the phosphate group is coordinated in place by R495_PL5_, while the arabinose moiety forms hydrogen bonds with E313_PL2_ and Q431_PL4_ which themselves are further stabilized by N304_PL2_ and Y320_PL2_ (Fig. 3D). W505_α6_ and T518 on PL5 also hold the DPA headgroup from the other side of the cavity (Fig. 3D). Most of the residues in this cavity are highly conserved amongst mycobacterial EmbB proteins (Fig. S8), and mutations within this hydrophilic cavity result in severe loss of the arabinosyltransferase activity (Figs. S5A and 5B). The last seven prenyl groups of DPA extend to the other half of the hydrophobic groove via extensive hydrophobic interactions with I421_TMH7_, V438_TMH8_, I448_TMH8_, I468_TMH9_ and I469_TMH9_ in the membrane space, with the end of the tail interacting with F670_TM14_ from the other EmbB protomer (Fig. 3E). Collectively, the PLs, PD_C_, TMH7, bended TMH8, TMH9 of the DPA-bound EmbB protomer and the TMH14 from the other protomer shape an ideal environment to accommodate DPA in the active state of the complex. Another structural rearrangement upon DPA binding, by comparing the structures of the two states, involves α6 in PL5, a short α helix that interacts with DPA, whereas in the resting state, this region is disordered (Fig. 3F). Considering that the bound DPA is the major composition difference between the two states, it is reasonable to speculate that the dramatic structural rearrangement of the enzyme complex in the active state occurs upon DPA binding, and is mediated by the induced disorder-to-helix transition of α6 at the dimer interface. This kind of concerted disordered-to-helix transition of a functional periplasmic loop along with donor binding has been proposed to enable access to the acceptor in other glycosyltransferases such as ArnT from *Cupriavidus metallidurans* in complex with its lipid substrate undecaprenyl phosphate (UndP) and PglB from *Campylobacter lari* with lipid-linked oligosaccharide (LLO) (Lizak et al., 2011; Vasileios I. Petrou et al., 2016; Napiorkowska et al., 2017).

### EmbB-AcpM interaction

**Figure 4:**
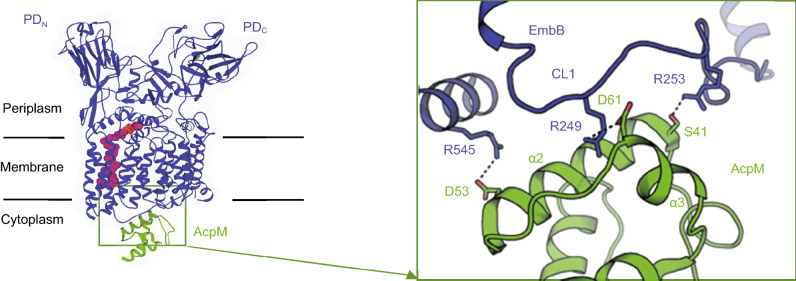
**EmbB-AcpM interaction and implications of by-product recycling**. Interface of EmbB-AcpM interaction, AcpM has a four-helix topology. Three pairs of residues participate in the EmbB-AcpM interaction, side-chains are shown as sticks

AcpM has a four-helix topology arranged in a right-handed bundle held together by interhelical hydrophobic interactions (Wong et al., 2002) (Fig. 2C). The two AcpM molecules bind to each of the two EmbB protomers through extensive electrostatic interactions on the cytoplasmic surface of EmbB (Fig. 4A). Helix-2 and helix-3 of AcpM are intimately engaged with the cytoplasmic loop 1 (CL1) of EmbB, which is a long positively charged linker between TMH2 and TMH3 (Fig. 4A). This is consistent with the known role of helix-2 in AcpM as a contact site with its target proteins, such as AcpS (Parris et al., 2000) (Fig. S6). The AcpM attached to the DPA-bound EmbB protomer has the better-resolved structure in our cryo-EM reconstructions (Figs. S2C, S2F, and S4B). Most of its side-chains have been assigned (Table S2). Three pairs of interactions are revealed: D53 and D61 of AcpM form salt-bridges with R454_TMH8b_ and R249_CL1_ of EmbB, and S41_AcpM_ forms a hydrogen bond with R253_CL1_ of EmbB (Fig. 4A). R249_CL1_, R253_CL1_, and R454_TMH8b_ in EmbB are highly conserved across mycobacterial species (Fig. S8). When a triple-alanine mutant (R249, R253, and R454) of EmbB that abolished its interaction with AcpM (Fig. S1C) was transformed into *Msm embB* knockout strain, a moderate loss (at ~40%) of the 3-arm branching signal of AG was observed (Fig. S1G), suggesting that AcpM is most likely functionally associated to arabinosyltransferase activity *in vivo*, regardless the equivalent mutation had little effect on the cell-free arabinosyltransferase activity (Fig. S5A).

### Active site and possible acceptor pathway

**Figure 5:**
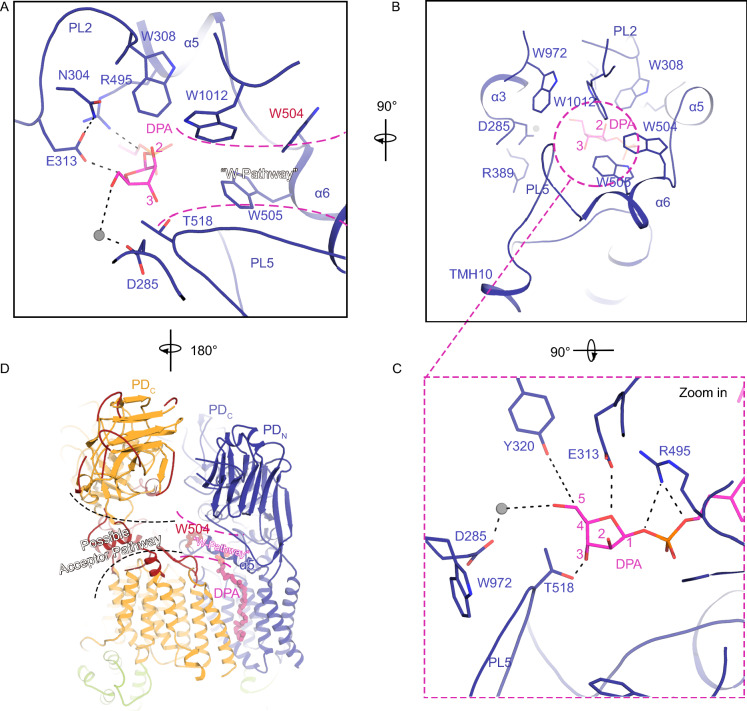
**Active site and possible acceptor pathway**. (A) DPA bound cavity viewed from PD_C_. An open path (W-pathway) between W1012 and W505 connecting to the DPA arabinose moiety at the dimer interface is indicated by the dashed curves. Surrounding side-chains interacting with DPA are shown as stick models. (B) Orthogonal view of (A) with the W-pathway to the DPA arabinose moiety is indicated by a dashed circle. (C) Orthogonal and zoom-in view of (B). (D) Flexibility of PLs in the empty protomer suggests an extending of the presumed acceptor approaching pathway in panel (A) which is indicated by W504 at the entrance. See also Fig. S3E

Glycosyltransferases are classified as either “inverting” or “retaining” based on two stereochemical outcomes in the formation of the new glycosidic bond at the anomeric carbon (Lairson et al., 2008). Emb proteins are believed to be inverting enzymes based on the fact that nearly all of the arabinose residues in the DPA-involved extension of AG are in an α-configuration (Jankute et al., 2015) (except for the non-reducing end of AG catalyzed by AftB (Seidel et al., 2007)), while DPA is in a β-configuration (Lee et al., 1995). For a conventional inverting glycosyltransferase reaction, upon both substrates (the donor and acceptor) reaching the catalytic site, a key catalytic step relies on an active site side-chain, which serves as a base catalyst that deprotonates the nucleophile of the acceptor, enabling the following nucleophilic attack at the anomeric carbon (Breton et al., 2012). In our structure, the arabinose moiety of DPA is positioned between two acidic residues D285 and E313 in the catalytic pocket (Fig. 3D). D285 belongs to the highly conserved DDx GT-C motif and is the predicted catalytically essential residue (Berg et al., 2005). Mutation of this residue in EmbB in this study and the equivalent residue D279 in EmbC as previously reported (Berg et al., 2005) led to a complete loss of activity (Fig. S5B). In EmbB, D285 locates at the junction of two cavities, one of which harbors the arabinose moiety and phosphate group of DPA, and the other, opposite to the DPA cavity, provides polar access to the dimer interface above the membrane boundary (Figs. 5A, 5B, and 5D). Altogether, this suggests that D285 is likely the key active site residue involved in catalysis. In our structure, DPA aligns its C5 atom close to D285, whereas the C1 atom is approximately 8 Å from the side-chain of D285. It is speculated that a further conformational change is required, possibly upon acceptor binding, allowing the DPA to move deeper into the cavity and reorient its C1 atom for α(1→3) glycosidic bond formation. This shift could involve a repositioning of the phosphate group much deeper into the DPA cavity and closer to D285.

EmbB catalyzes the penultimate step of AG synthesis. We have previously reported that EmbB functions in a coordinating way with EmbA (Zhang et al., 2020), nevertheless, our cell-free assay has confirmed that purified EmbB alone is functional using NV6 as acceptor. Thus, it can be inferred that EmbB may play dual roles in cells, by either forming a homodimer or a heterodimer with EmbA, depending on the physiological concentration of these proteins. Therefore, the native acceptor of EmbB could be a relatively mature arabinan chain before the terminal 3-arm branch of the characteristic hexa-arabinan motif (Fig. 1A). The possible acceptor entry pathway could be deduced based on the location of the identified donor binding cavity and the putative active site. This polar pathway as mentioned above is a tryptophan-rich region that we refer to as the “W-pathway” (Fig. 5A). This W-pathway is formed by a series of tryptophan residues W308, W504, W505, W972, and W1012. The first three tryptophan residues, located on the DDX-motif-containing PL2 and conformational change modulator PL5, extend to the arabinose moiety of DPA, while the latter two tryptophan residues are located on the mixed α/β fold subdomain of PD_C_. The role of W972 was previously reported to be critical in terms of both of the binding affinity with acceptor and the enzymatic activity in EmbC (W985 in EmbC corresponds to W972 in EmbB) (Alderwick et al., 2011). These tryptophan residues are only partially conserved amongst EmbA, EmbB, and EmbC. The differences might reflect the fact that Emb proteins are different enzymes with different substrate specificities. Furthermore, previous studies have attributed the C-terminal domain of the Emb proteins to a critical role in acceptor substrate recognition and arabinan chain extension (Shi et al., 2006; Alderwick et al., 2011). Consistent with this, in the active state structure, several regions of the PD_C_ are missing in the DPA unbound EmbB protomer (Figs. 5D and S3E). The flexibility of these missing regions could be the result of the absence of the acceptor when the EmbB_2_-AcpM_2_ complex is activated by the donor. Consequently, this region in PDc may outline a fairly broad and open pathway leading straight into the W-pathway headed by W504 which locates in the dimer interface (Fig. 5D), thus indicating a macroscopic acceptor entry pathway.

### Proposed catalytic cycle for the arabinosyltransferase complex

In this study, we have determined the structures of a substrate unbound EmbB_2_-AcpM_2_ complex in a presumed resting state and a donor DPA-bound pre-catalytic conformation which we define as the “active” state. The two structures of EmbB_2_-AcpM_2_ suggest that the binding of substrates is a sequentially coupled process with substantial conformational changes upon the binding of the two substrates.

**Figure 6:**
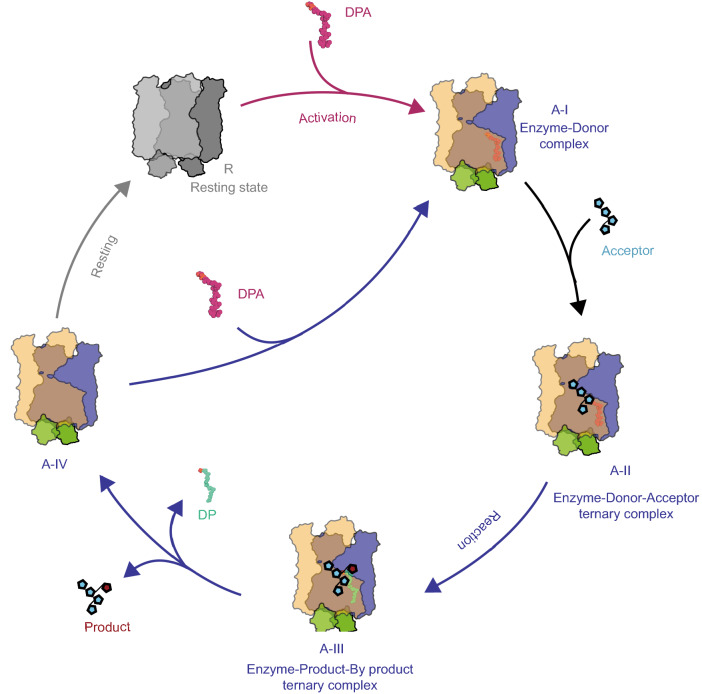
**A proposed catalytic cycle for the arabinosyltransferase complex EmbB**_**2**_**-AcpM**_**2**_. The components of the complex are colored as in Fig. 1B. The arabinofuranosyl motif of the donor (tetraarabinan) and product (pentaarabinan) are depicted as a chain of cyan pentagons (the arabinose moiety transferred from DPA is in red). Donor (DPA) and by-product (DP) molecules shown as spheres in their different locations are colored as Fig. 4A and 4B. The bold arrow lines in grey, red and blue indicate the resting state, activation and processing states during turn-over. Dashed arrow lines indicate a possible recycle mechanism of DP through a switching of DP-carrying AcpM and a hollow one. Different states in the active phase are labeled as A-I, A-II, A-III, A-IV

We hypothesize that DPA binding activates the EmbB dimer by triggering conformational changes. Once the EmbB_2_-AcpM_2_ complex is activated, the glycosyltransferase reaction can occur in the following defined steps (Fig. 6).

A-I. The acceptor is recognized and threaded by PD_C_ and enters the active site through the W-pathway, forming an enzyme-donor-acceptor ternary complex (A-II) ready for the reaction. The DPA bound EmbB_2_-AcpM_2_ structure in this study represents an intermediate state before C1 of DPA reaches the optimal position for catalysis. Hence, to form the ternary complex a series of precisely arranged conformational changes are required to allow both substrates to be oriented optimally.

A-II. Based on the catalytically essential role of D285 and canonical mechanism of an inverting glycosyltransferase (Qasba et al., 2005; Lairson et al., 2008), the following is rationally proposed: D285 deprotonates the hydroxyl group on the C3 carbon of the acceptor and activates this hydroxyl group for a nucleophilic attack on the C1 carbon of DPA. A new glycosidic bond is formed (A-III) allowing the newly formed arabinan product and DP (the leaving group) to release from the active site.

A-III. Overall, functional studies confirm that EmbB catalyzes arabinose chain α(1→3) branching with the subsequent product utilized by AftB for the terminal β(1→2) linkage at the non-reducing end of AG. Therefore, it is plausible that product release (A-IV) could be coupled instantaneously with a new round of DPA binding (A-I).

### Structural mapping of ethambutol resistance associated mutations and its potential binding site

**Figure 7:**
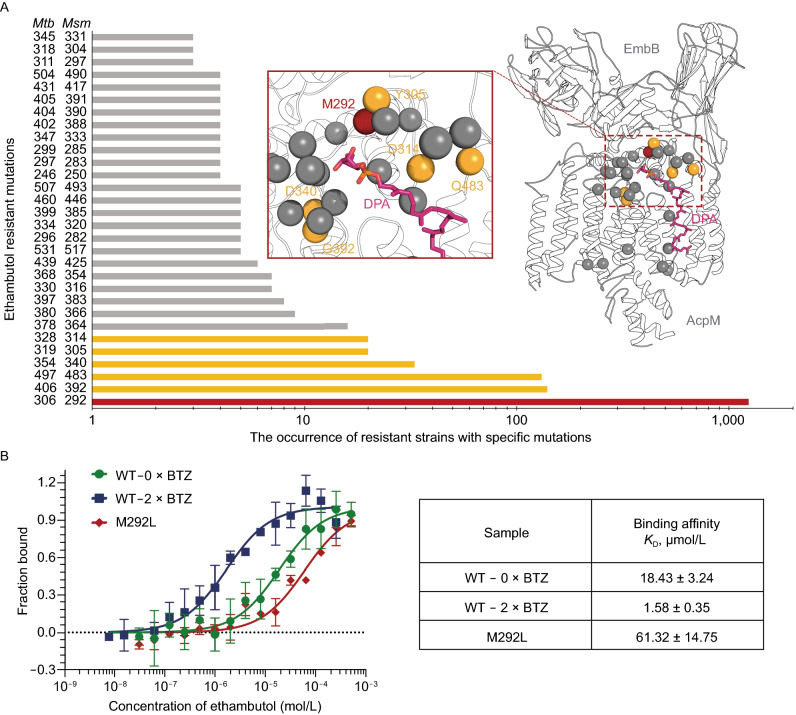
**Mapping of ethambutol-resistant mutation sites**. (A) Log-Scaled histogram of the occurrence of the top 30 frequent ethambutol-resistant mutations in EmbB in 61 studies collected from the drug-resistance database, MycoResistance, and their positions on the DPA-bound EmbB structure, most notably around DPA binding cavity. Mutations with more than 20 occurrences are colored in yellow, and those with more than 1,000 occurrences are in red. (B) MST assays for the binding of ethambutol to wild-type EmbB treated with 2× MIC of BTZ-043 or inhibitor-free during culture and ethambutol resistant mutation hotspot M292L represented by red sphere in (A). Error bars represent mean ± SEM based on three independent measurements. Binding curves and *K*_D_ values are also shown. See also Figs. S7 and S8

In this study, we have shown that anti-TB drug ethambutol inhibits the α(1→3) arabinosyltransferase activity of purified EmbB protein. This agrees with the fact that branching of the terminal hexaarabinan motif in AG can be inhibited by ethambutol (Lee et al., 2005). Numerous mutations in *embB* are causally associated with resistance to ethambutol, but their effects on EmbB structure and function remain unclear. The three-dimensional structure of EmbB provides an unprecedented opportunity to understand the role that the *embB* mutations play in the development of ethambutol resistance in *M*. *tuberculosis*. We selected the top 30 most frequent ethambutol-resistant mutations of EmbB in 1,814 strains from 61 studies in a manually collated drug-resistance database MycoResistance (Dai et al., 2019) (Figs. 7A and S8). Upon mapping these onto the DPA-bound EmbB structure, we find that most of the mutations concentrate in a radial region centered on the DPA-bound pocket (Fig. 7A). M306 (equivalent to *Msm* M292) in *Mtb* EmbB which is the most common clinical mutation associated with ethambutol resistance (Ramaswamy et al., 2000; Lee et al., 2005; Safi et al., 2008) is, as expected, also the most predominant (1243/2353) in our analysis (Fig. 7A). Clinical isolate mutations include M306L, M306V, M306I and M306T substitutions in *Mtb* (Lee et al., 2002; Sun et al., 2018). In *Msm*, M292T mutation results in a 60-fold increase in MIC (Lety et al., 1997). In our cell-free enzymatic activity assay, mutein M292L demonstrates clear resistance to ethambutol (Fig. S5A). Meanwhile, the binding affinity of ethambutol to this mutein also significantly decreases (Fig. 7B).

The recently reported structure of the ethambutol bound EmbA-EmbB complex enables us to analyze the structural features of the potential drug binding pockets of EmbB2 in this study. When superimposing the EmbB protomer from the EmbB2 in its resting state onto the EmbB subunit of ethambutol-bound EmbA-EmbB complex (Zhang et al., 2020), residues including the catalytic site D285 on EmbB2 were obsereved in the similar position (Figure S7A). Thus it is possible the ethambutol inhibition mode on EmbB2 similar to that on EmbA-EmbB complex by inhibiting substrate binding. Notable shifting (2.9~4.0 A°) were observed on E313 on PL2 and H580 on PL6 (Figure S7A), likely due to ethambutol binding. The binding affinity of EmbB with ethambutol is greatly enhanced (*K*_D_ = 1.58 μmol/L) (Fig. 7B) when a DPA synthesis inhibitor, BTZ043 (Makarov et al., 2009), was added during cell culture to obtain overexpressed EmbB protein in a relatively low-abundance DPA environment, which could support this speculation. The competitive inhibition hypothesis of ethambutol is also in agreement with a previous DPA recognition study which demonstrated a rapid accumulation of DPA in ethambutol treated Msm cells(Wolucka et al., 1994).

These analyses will facilitate further experimental studies aimed at understanding how mutations in EmbB and other Emb proteins lead to ethambutol resistance. To be noted, not all ethambutol-resistant strains have *embB* mutations (Alcaide et al., 1997), suggesting that further studies on other resistance associated proteins or pathways are necessary to complete a comprehensive understanding of the mechanisms of ethambutol resistance.

## Conclusions

In this study, cryo-EM single-particle analysis revealed conformational heterogeneity in arabinosyltransferase complex EmbB_2_-AcpM_2_ which we ascribe to evidence for a “resting” and “donor-bound active” state. An acceptor recognition and entry pathway is proposed. We have also characterized EmbB as an α(1→3) arabinosyltransferase and confirmed it is a target of ethambutol. Thus, our work not only unravels the molecular mechanisms involved in substrate recognition for this arabinosyltransferase but also provides a much-anticipated foundation for developing improved anti-TB drugs such as inert DPA analogues.

## Electronic supplementary material

Below is the link to the electronic supplementary material.

Supplementary material 1 (PDF 3993 kb)

## References

[CR1] Alcaide F, Pfyffer GE, Telenti A (1997). Role of embB in natural and acquired resistance to ethambutol in mycobacteria. Antimicrob Agents Chemother.

[CR2] Alderwick LJ, Lloyd GS, Ghadbane H, May JW, Bhatt A, Eggeling L, Futterer K, Besra GS (2011). The C-terminal domain of the arabinosyltransferase *Mycobacterium tuberculosis* EmbC is a lectin-like carbohydrate binding module. PLoS Pathog.

[CR3] Alliance T (2008). Ethambutol. Tuberculosis.

[CR5] Berg S, Starbuck J, Torrelles JB, Vissa VD, Crick DC, Chatterjee D, Brennan PJ (2005). Roles of conserved proline and glycosyltransferase motifs of EmbC in biosynthesis of lipoarabinomannan. J Biol Chem.

[CR4] Berg S, Kaur D, Jackson M, Brennan PJ (2007). The glycosyltransferases of *Mycobacterium tuberculosis*—roles in the synthesis of arabinogalaetan, lipoarahinolrnlannan, and other glyeoeonjugates. Glycobiology.

[CR6] Breton C, Fournel-Gigleux S, Palcic MM (2012). Recent structures, evolution and mechanisms of glycosyltransferases. Curr Opin Struct Biol.

[CR7] Dai E, Zhang H, Zhou X, Song Q, Li D, Luo L, Xu X, Jiang W, Ling H (2019). MycoResistance: a curated resource of drug resistance molecules in *Mycobacteria*. Database.

[CR8] Escuyer VE, Lety MA, Torrelles JB, Khoo KH, Tang JB, Rithner CD, Frehel C, McNeil MR, Brennan PJ, Chatterjee D (2001). The role of the embA and embB gene products in the biosynthesis of the terminal hexaarabinofuranosyl motif of *Mycobacterium smegmatis* arabinogalactan. J Biol Chem.

[CR9] Goude R, Amin AG, Chatterjee D, Parish T (2008). The critical role of embC in *Mycobacterium tuberculosis*. J Bacteriol.

[CR10] Jankute M, Cox JA, Harrison J, Besra GS (2015). Assembly of the mycobacterial cell wall. Annu Rev Microbiol.

[CR11] Lairson LL, Henrissat B, Davies GJ, Withers SG (2008). Glycosyltransferases: structures, functions, and mechanisms. Annu Rev Biochem.

[CR15] Lee RE, Mikusova K, Brennan PJ, Besra GS (1995). Synthesis of the mycobacterial arabinose donor beta-d-arabinofuranosyl-1-monophosphoryldecaprenol, development of a basic arabinosyl-transferase assay, and identification of ethambutol as an arabinosyl transferase inhibitor. J Am Chem Soc.

[CR13] Lee RE, Brennan PJ, Besra GS (1997). Mycobacterial arabinan biosynthesis: the use of synthetic arabinoside acceptors in the development of an arabinosyl transfer assay. Glycobiology.

[CR12] Lee HY, Myoung HJ, Bang HE, Bai GH, Kim SJ, Kim JD, Cho SN (2002). Mutations in the embB locus among Korean clinical isolates of *Mycobacterium tuberculosis* resistant to ethambutol. Yonsei Med J.

[CR14] Lee RE, Li W, Chatterjee D, Lee RE (2005). Rapid structural characterization of the arabinogalactan and lipoarabinomannan in live mycobacterial cells using 2D and 3D HR-MAS NMR: structural changes in the Arabinan due to ethambutol treatment and gene mutation are observed. Glycobiology.

[CR16] Lety MA, Nair S, Berche P, Escuyer V (1997). A single point mutation in the embB gene is responsible for resistance to ethambutol in *Mycobacterium smegmatis*. Antimicrob Agents Chemother.

[CR17] Lizak C, Gerber S, Numao S, Aebi M, Locher KP (2011). X-ray structure of a bacterial oligosaccharyltransferase. Nature.

[CR18] Makarov V, Manina G, Mikusova K, Mollmann U, Ryabova O, Saint-Joanis B, Dhar N, Pasca MR, Buroni S, Lucarelli AP (2009). Benzothiazinones kill *Mycobacterium tuberculosis* by blocking Arabinan synthesis. Science.

[CR19] Mikusova K, Slayden RA, Besra GS, Brennan PJ (1995). Biogenesis of the mycobacterial cell wall and the site of action of ethambutol. Antimicrob Agents Chemother.

[CR20] Napiorkowska M, Boilevin J, Sovdat T, Darbre T, Reymond JL, Aebi M, Locher KP (2017). Molecular basis of lipid-linked oligosaccharide recognition and processing by bacterial oligosaccharyltransferase. Nat Struct Mol Biol.

[CR21] Parris KD, Lin L, Tam A, Mathew R, Hixon J, Stahl M, Fritz CC, Seehra J, Somers WS (2000). Crystal structures of substrate binding to *Bacillus subtilis* holo-(acyl carrier protein) synthase reveal a novel trimeric arrangement of molecules resulting in three active sites. Structure.

[CR22] Qasba PK, Ramakrishnan B, Boeggeman E (2005). Substrate-induced conformational changes in glycosyltransferases. Trends Biochem Sci.

[CR23] Ramaswamy SV, Amin AG, Goksel S, Stager CE, Dou SJ, El Sahly H, Moghazeh SL, Kreiswirth BN, Musser JM (2000). Molecular genetic analysis of nucleotide polymorphisms associated with ethambutol resistance in human isolates of *Mycobacterium tuberculosis*. Antimicrob Agents Chemother.

[CR24] Safi H, Sayers B, Hazbon MH, Alland D (2008). Transfer of embB codon 306 mutations into clinical *Mycobacterium tuberculosis* strains alters susceptibility to ethambutol, isoniazid, and rifampin. Antimicrob Agents Chemother.

[CR25] Sassetti CM, Boyd DH, Rubin EJ (2003). Genes required for mycobacterial growth defined by high density mutagenesis. Mol Microbiol.

[CR26] Seidel M, Alderwick LJ, Birch HL, Sahm H, Eggeling L, Besra GS (2007). Identification of a novel arabinofuranosyltransferase AftB involved in a terminal step of cell wall arabinan biosynthesis in *Corynebacterianeae*, such as *Corynebacterium glutamicum* and *Mycobacterium tuberculosis*. J Biol Chem.

[CR27] Shi L, Berg S, Lee A, Spencer JS, Zhang J, Vissa V, McNeil MR, Khoo KH, Chatterjee D (2006). The carboxy terminus of EmbC from *Mycobacterium smegmatis* mediates chain length extension of the arabinan in lipoarabinomannan. J Biol Chem.

[CR28] Snapper SB, Melton RE, Mustafa S, Kieser T, Jacobs WR (1990). Isolation and characterization of efficient plasmid transformation mutants of *Mycobacterium smegmatis*. Mol Microbiol.

[CR29] Sun Q, Xiao TY, Liu HC, Zhao XQ, Liu ZG, Li YN, Zeng H, Zhao LL, Wan KL (2018). Mutations within embCAB are associated with variable level of ethambutol resistance in *Mycobacterium tuberculosis* isolates from China. Antimicrob Agents Chemother.

[CR30] Takayama K, Kilburn JO (1989). Inhibition of synthesis of arabinogalactan by ethambutol in *Mycobacterium smegmatis*. Antimicrob Agents Chemother.

[CR31] Vasileios I, Herrera CM, Schultz KM, Clarke OB, Vendome J, Tomasek D, Banerjee S, Rajashankar KR, Dufrisne MB, Kloss B (2016). Structures of aminoarabinose transferase ArnT suggest a molecular basis for lipid A glycosylation. Nature.

[CR32] WHO (2018). Global tuberculosis report 2018.

[CR33] Wolucka BA, McNeil MR, de Hoffmann E, Chojnacki T, Brennan PJ (1994). Recognition of the lipid intermediate for arabinogalactan/arabinomannan biosynthesis and its relation to the mode of action of ethambutol on mycobacteria. J Biol Chem.

[CR34] Wong HC, Liu G, Zhang YM, Rock CO, Zheng J (2002). The solution structure of acyl carrier protein from *Mycobacterium tuberculosis*. J Biol Chem.

[CR35] Zhang L, Zhao Y, Gao Y, Wu L, Gao R, Zhang Q, Wang Y, Wu C, Wu F, Gurcha SS (2020). Structures of cell wall arabinosyl transferases with the anti-tuberculosis drug ethambutol. Science.

