# Supplementary material for: Cryo-EM snapshots of mycobacterial arabinosyltransferase complex EmbB2-AcpM2
Source: Protein Cell. 2020 May 3;11(7):505–17. doi: 10.1007/s13238-020-00726-6 (PMC7305291; doi:10.1007/s13238-020-00726-6)
Supplement: Supplementary file 1 — Supplementary material 1 (PDF 3993 kb) [file 13238_2020_726_MOESM1_ESM.pdf]

METHOD DETAILS

Protein expression and membrane preparation

The *embB* gene (*MSMEG_6389*) from *M. smegmatis* strain mc^{2}155 genomic was cloned into the pMV261 vector fused to a 10 × His tag at the C-terminal of the EmbB, under the control of an acetamide promoter. Recombinant *Msm*-EmbB was transformed into *Msm* mc^{2}155 competent cells by electroporation. Cells were cultured in large scale in 1 L Luria-Broth (LB) medium supplemented with 50 μg/mL kanamycin, 20 μg/mL carbenicillin, and 0.1% (v/v) Tween80 (to avoid cell aggregation) at 37 °C with shaking at 220 rpm until the OD_{600} reached 1.0. Four days after induction with 0.2% (w/v) acetamide at 16 °C, the cells were collected in Buffer A containing 20 mM HEPES,150 mM NaCl, 2 mM DTT and 5% (v/v) glycerol, pH 7.4, and lysed by passing through a French Press at 1,200 bar at 4 °C. Cell debris was cleared by centrifugation at 10000 g for 10 min at 4 °C. The membrane pellet was collected by ultracentrifugation (150,000 g, 1 h) at 4 °C then resuspended in Buffer A and stored at –80 °C until use. All mutants were expressed using the same protocol as the wild-type protein.

Protein purification and preparation for cryo-EM

Thawed membrane fraction was solubilized with 1% *n*-undecyl-β-D-maltopyranoside (UDM; Anatrace) by gently agitating for 1.5h at 4 °C. Detergent-insoluble material was removed by ultracentrifugation (18,000 rpm, 30 min). Supernatant was purified by TALON metal affinity resin (Clontech) followed by size-exclusion chromatography using a Superose 6 Increase column (GE Healthcare) pre-equilibrated with Buffer B containing 20 mM HEPES, 150 mM NaCl, pH 7.4, and UDM at the twice critical micellar concentration (CMC). Before amphipol exchange, the peak fraction was crosslinked with

0.2% glutaraldehyde at RT for 45 min. Crosslinking was terminated by addition of Tris-HCl and then the protein was mixed with PMAL-C8 (Anatrace) at a 1:3 (w/w) dilution with gentle agitation at 4 °C. Detergent was removed by incubation with 20 mg/mL Bio-Beads SM2 (Bio-Rad) overnight at 4 °C. The Bio-Beads were then removed and eluent cleared by centrifugation before further separation on a Superose 6 Increase column equilibrated in a detergent-free buffer to remove the residual detergent and free amphipol. The peak fraction corresponding to dimeric EmbB-AcpM (at ~0.4 mg/mL) was used directly for cryo-EM. It should be noted that for characterization of cell-free arabinosyltransferase activity, the crosslinking and amphipol exchange steps were omitted and the purified complex was stored in an assay buffer (Buffer C) containing 50 mM MOPS, 10 mM $MgCl_2$, pH 7.9, 5 mM β-mercaptoethanol, 5% (v/v) glycerol and *n*-dodecyl-β-D-maltopyranoside (DDM) at the twice CMC.

Grid preparation and data collection

Aliquots of the freshly purified sample were applied to glow-discharged holey carbon grids (Quantifoil Au R1.2/1.3). Grids were blotted for 2.5 s and flash-frozen in liquid ethane cooled by liquid nitrogen using an FEI Mark IV Vitrobot. Images were taken using an FEI Titan Krios electron microscope operating at 300 kV with a Gatan K2 Summit detector at a nominal magnification of 165kx. Images were recorded in the super-resolution mode and binned to a pixel size of 0.82 Å. Automated single-particle data acquisition was performed with SerialEM (Mastronarde, 2003). Defocus values varied from 1.5 to 2.5 μm. Each stack was exposed for 6.8 s or 5.1 s with a total dose of 60 e/Å^2, with 40 frames or 32 frames per stack in each different collection batch.

EM image processing

Among all raw cryo-EM stacks, 14,694 micrographs were generated by MotionCor2 (Zheng et al., 2017), of which around 2,382 k particles were initially picked out by Gautomatch (http://www.mrc-lmb.cam.ac.uk/kzhang/Gautomatch). From those particles, a series of 2D/3D classifications of RELION 2.1 (Kimanius et al., 2016; Scheres, 2012a, b) were conducted aiming to fully purify and extract suitable candidates for further refinement, around 349 k particles were left and thus subjected to further refinement, resulting in a dimer map at 4.2 Å (according to gold-standard FSC), and a protomer map of 3.9 Å with a handcrafted "half-mask" applied. All motion correction and dose-weighting procedures were performed by MotionCor2 while contrast transfer function calculation and local ctf estimation during the later stage were performed by Gctf (Zhang, 2016).

During 3D classification, it was observed that around 10% of particles exhibited a different dimer conformation compared to the rest, thus around 3,284 K particles out of 19,612 micrographs from all available stacks were assembled and subjected to a new round of processing (Figure S2) and 1,293 K particles were thereby selected after the cascade of classifications in RELION 2.1, for particles in the two conformations. Subsequently, with initial models from previous runs in RELION 2.1, RELION refinement and further 3D classification (skip-alignment) were performed, from which two groups of particles were selected. These two groups of particles were then fed to cryoSPARC (Punjani et al., 2017) for refinement respectively, yielding an asymmetric dimer map of 3.5 Å and a pseudo-C2 dimer map of 3.6 Å. For better visualization, maps

were sharpened by the introduction of negative B-factors (Rosenthal and Henderson, 2003). Local resolution variations were estimated by cryoSPARC.

Model building and refinement

All residues for each protomer were set to alanine in the initial build and assigned later guided by secondary structure prediction of Phyre2 (Kelley et al., 2015). The protomers were then docked with Chimera (Pettersen et al., 2004) to form the EmbB dimers but with two different conformations, and whose models were later refined in Coot 0.8 (Emsley et al., 2010), while the periplasmic domain at C-terminus of the empty protomer in "active state" dimer was docked using the 3.9 Å EM map instead of the 3.5 Å map, due to its flexibility-caused shatter in this region. The AcpM homology model was obtained from the PDB with code 1klp and docked into the cryo-EM map using Chimera and then refined in Coot 0.8. All models were later refined in PHENIX (Adams et al., 2010) and REFMAC5 (Murshudov et al., 2011), LocalDeblur (Ramirez-Aportela et al., 2018) was used for the 3.5 Å EM map for enhanced interpretability. Details are shown in Table S2.

Transferase activity assay

Arabinosyltransferase assays were essentially performed as described previously (Lee et al., 1997) using NV6 or NV13 (1 mM in water), DP[^{14}C]A (100,000 cpm, stored in 1% IgePal), 1 mM ATP, 1 mM NADP, EmbB proteins (4 μM) or *Msm* membrane and P60 fractions (1 mg each) and in some cases ethambutol, with the appropriate amount of buffer (50 mM MOPS, pH 7.9, 5 mM β-mercaptoethanol). All samples were made up to a final volume of 80 μL. These were incubated at 37 °C for 1 hour, quenched by the addition of 533 μL of chloroform/methanol (1:1, v/v) and mixed overnight at 4 °C. The supernatant was recovered following centrifugation and dried. The residue was resuspended in 2 mL

of ethanol/water (1:1, v/v) and loaded onto a 1 mL SAX SepPak and washed with 2 mL of ethanol and the eluate collected and dried. The sample was resuspended in a mixture of water-saturated n-butanol (2 mL) and water (2 mL) and the organic phase recovered. The aqueous phase was re-extracted using water-saturated n-butanol (2 mL) and the organic phases pooled and re-washed with water (2 mL). The organic layer was dried and resuspended in n-butanol. The incorporation of [^{14}C]arabinose from DP[^{14}C]A was determined by scintillation counting and by subjecting samples to TLC using silica gel plates developed in chloroform/methanol/water/ammonium hydroxide (65:25:3.6:0.5, v/v/v/v) and visualized by autoradiography using Kodak BioMAx MR films. Each assay was repeated three times.

Mass spectrometry

50 µL DDM purified EmbB$_2$-AcpM$_2$ protein was treated with 350 µL chloroform/methanol (1:1) then left overnight on ice. The suspension was converted to a bilayer by adding 250 µL chloroform/water (7:3) the next day. The lower organic phase was pooled after centrifugation and then dried in a speed vacuum concentrator. The dried lipids were re-dissolved in 20 µL chloroform/methanol. 1 µL of the sample was injected into QTOF (SCIEX 4600) MS coupled with UPLC (Shimadzu, 30A). After loading the sample onto the chromatography column (Waters Bioresolve Polyphenyl, 450 Å, 2.7 um, 2.1 × 150 mm) the product was eluted by gradient as follows: Buffer A (0.1% formic acid 1% acetonitrile) for 1 min, then 5% to 95% Buffer B (0.1% formic acid in acetonitrile) for 3 min, then 95% Buffer B for 3.5 min. The flow rate was 50 µL/min. The mass spectrometer was operated in negative mode. The source voltage, the curtain gas, and the source temperature were set to 4500 V, 30 psi and 350 °C respectively. A SIM scan

(m/z: 909.6, window width: 2 da for DPA and m/z: 777.6, window width: 2 da for DP) followed by a MS2 scan was used to detect the targeted lipid. The collision energy was set to 35 eV.

Molecular docking

Induced fit docking of the ethambutol to the EmbB protein was performed using Schrodinger (Friesner et al., 2004). Prior to docking, the protein structure was extracted from the complex structure, and was then prepared using the protein preparation wizard module. Protein was pre-processed, optimized and minimized using OPLS 2005 force field (Banks et al., 2005). All ligands were prepared through LipPrep module and all possible states at pH 7 ± 2 were generated using ionizer and retaining specific chiralities of the molecules. At most, 1000 conformations were generated per ligand molecule. Initially Glide docking was carried out for each ligand. The sample ring conformations of ligands were selected and the side-chains were trimmed. The prime side-chain prediction and minimization was carried out in which residues were refined within 6.0 Å of ligand poses and side-chains were optimized. This led to creation of a ligand structure and conformation that is induced-fit to each pose of the protein structure. Finally, Glide redocking was carried out using default conditions. The ligand was rigorously docked into the induced-fit protein structure and the results yielded by Glide score for each output pose.

MST assay

The assay was accomplished according to the previously reported method (Zhang et al., 2019). The binding affinity of the detergent purified EmbB for ethambutol was measured using a Monolith NT.115 (Nanotemper Technologies). The His-tagged protein

was labeled with RED-tris-NTA Dye according to the manufacturer’s procedure. For each assay, the labeled protein at 200 nM was incubated with the same volume of unlabeled ligands at 16 different concentrations in the same buffer as the protein at room temperature for 10 min. The samples were then loaded into capillaries (Nanotemper Technologies) and measured at 25 °C by using 40% LED and medium MST power. Each assay was repeated three times. K_D values were calculated using the MO. Affinity Analysis v.2.2.4 software. All of the final plots were made using GraphPad Prism 7.0.

Creation of figures

Figures of molecular structures were generated using PyMOL (The PyMOL Molecular Graphics System, Schrödinger, LLC.) (DeLano, 2010) and UCSF ChimeraX (Goddard et al., 2018).

QUANTIFICATION AND STATISTICAL ANALYSIS

The dissociation constants (K_D) in Microscale thermophoresis (MST) experiments were calculated using MO. Affinity Analysis v.2.2.4 software as the mean ± SEM from three independent experiments with a single site-specific binding model for EmbB proteins with ethambutol, and Hill equation mode for AcpM protein with phospholipids.

DATA AND SOFTWARE AVAILABILITY

All data are available in the manuscript or the supplementary materials. The accession no. for the 3D cryo-EM density maps reported in this paper is XXXX and XXXX. The PDB accession no. for the coordinates of the $EmbB_2$-$AcpM_2$ in DPA bound and unbound states are XXXX and XXXX.

Supplemental Figures

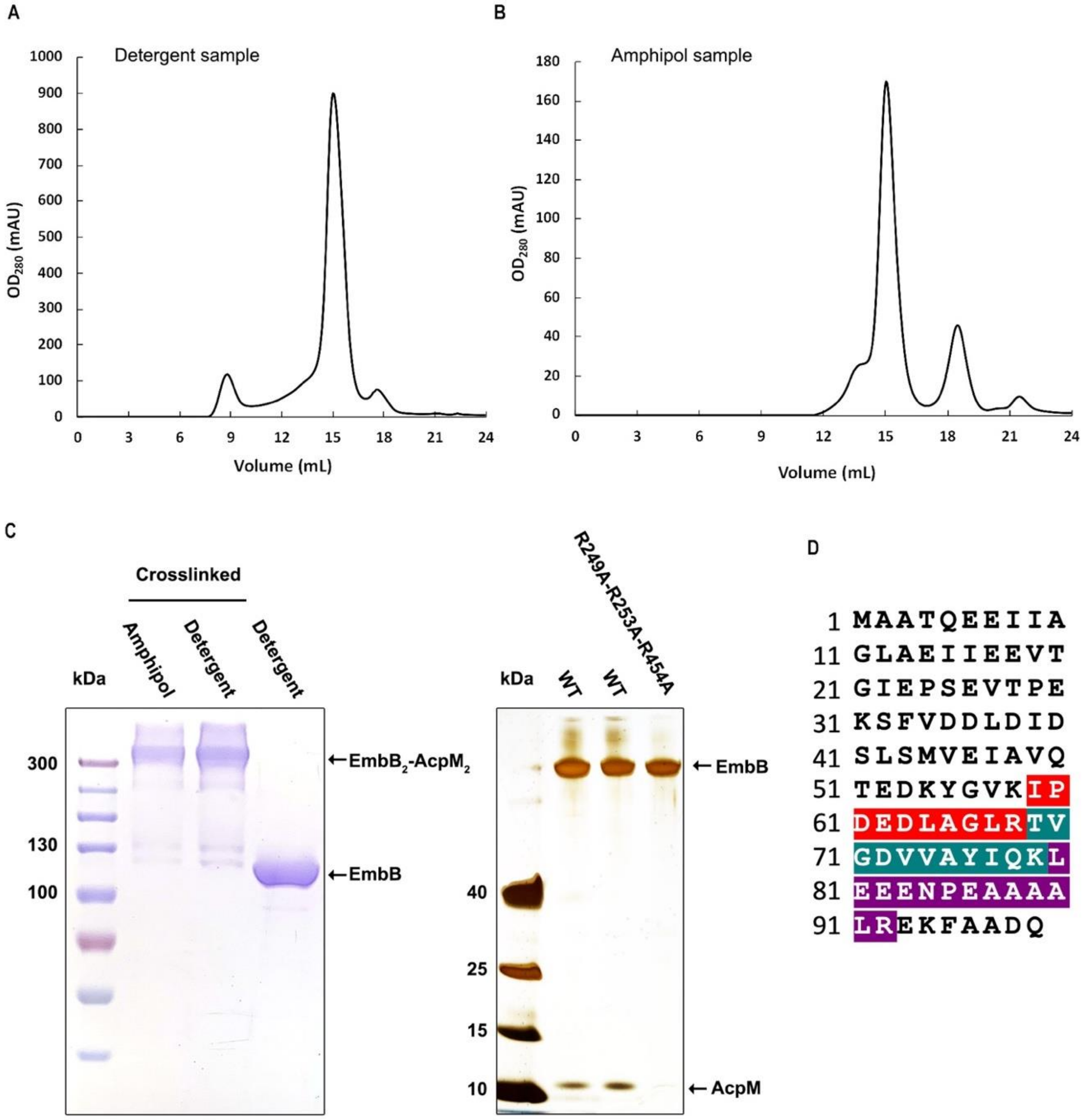

E

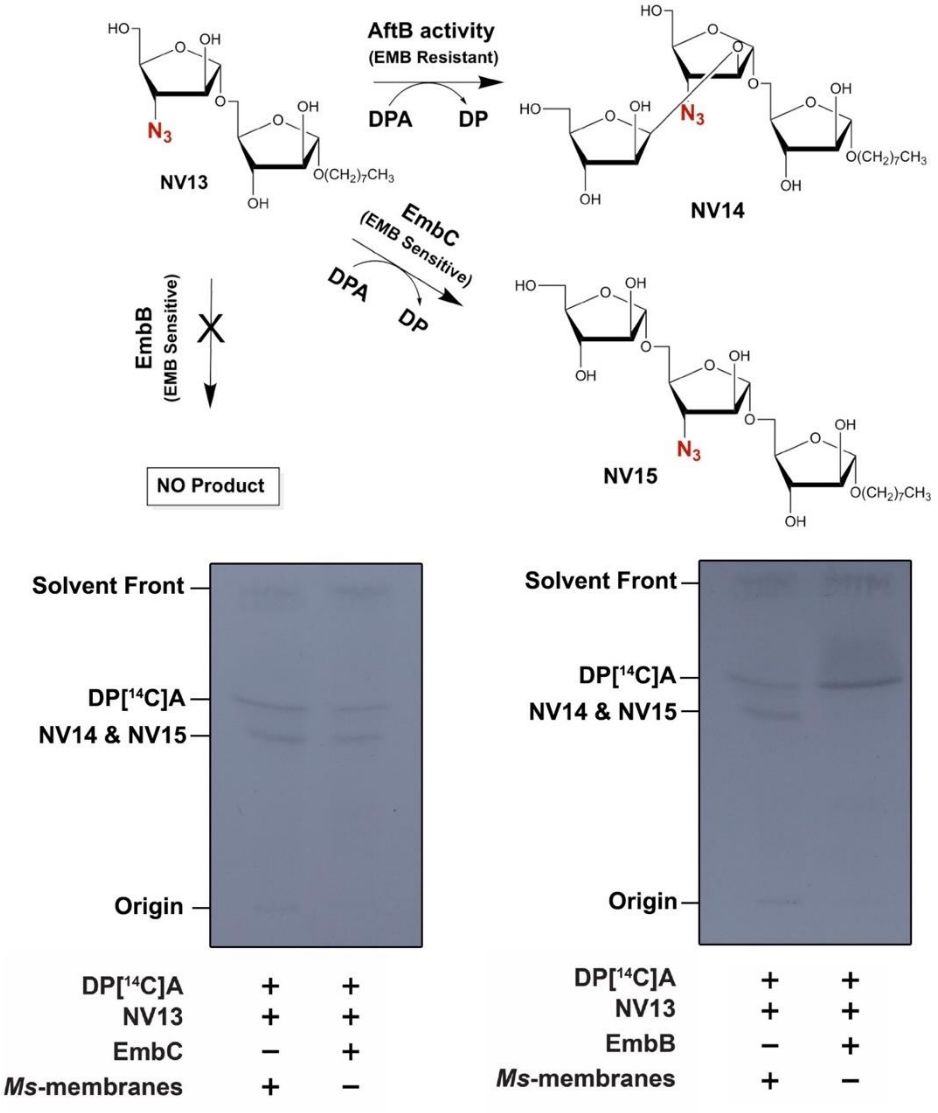
AftB activity
(EMB Resistant)
DPA
DP
NV13
NV14
EmbC
(EMB Sensitive)
DPA
DP
NV15
EmbB
(EMB Sensitive)
NO Product
Solvent Front
DP[14C]A
NV14 & NV15
Origin
DP[14C]A + +
NV13 + +
EmbC − +
Ms-membranes + −
Solvent Front
DP[14C]A
NV14 & NV15
Origin
DP[14C]A + +
NV13 + +
EmbB − +
Ms-membranes + −

F

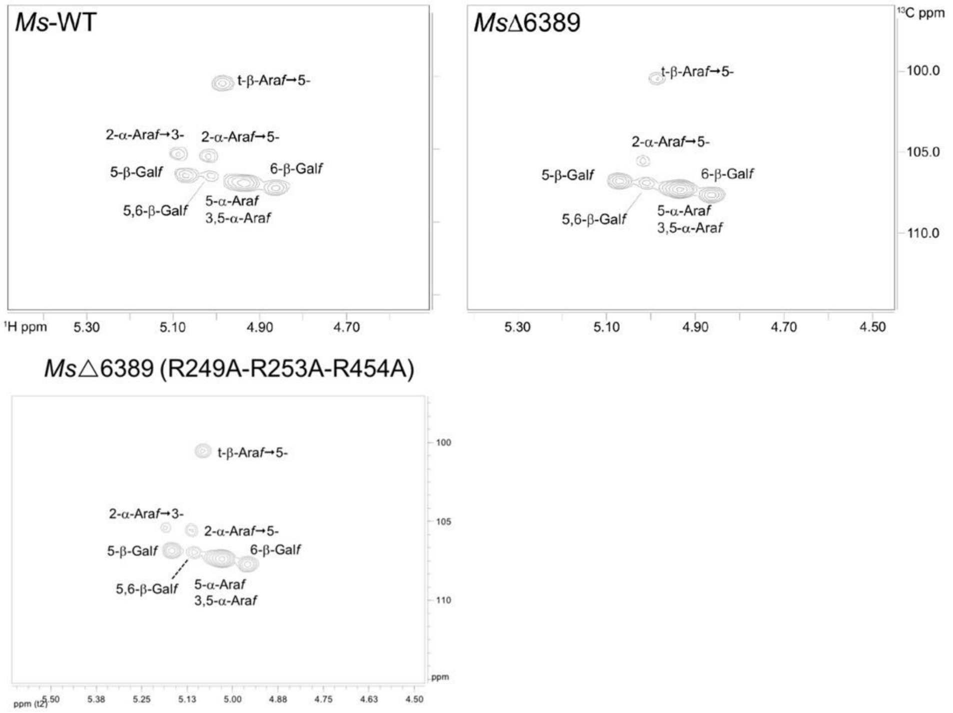
Ms-WT
MsΔ6389
Ms△6389 (R249A-R253A-R454A)
t-β-Araf→5-
2-α-Araf→3-
2-α-Araf→5-
5-β-Galf
6-β-Galf
5,6-β-Galf
5-α-Araf
3,5-α-Araf
13C ppm
1H ppm
ppm (t2)

G

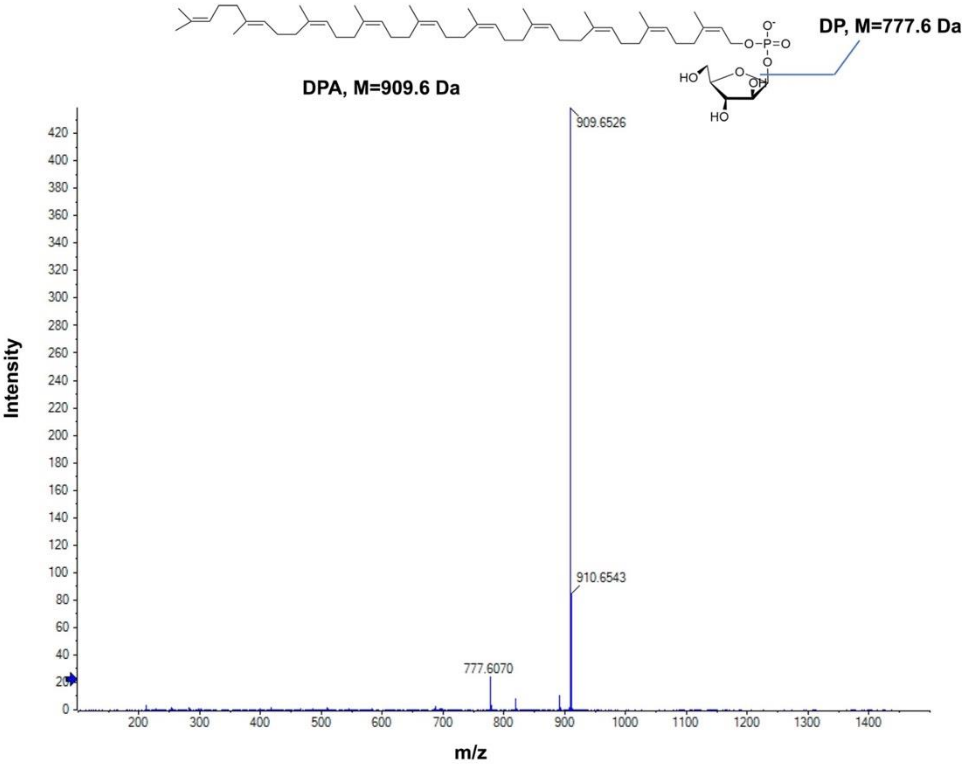
DP, M=777.6 Da
DPA, M=909.6 Da
909.6526
910.6543
777.6070
Intensity
m/z

165

Figure S1. Enzyme purification and characterization, related to Figures 1 and 3.

Size-exclusion chromatography of $EmbB_2$-$AcpM_2$ in detergent (**A**) and amphipol (**B**). (**C**) SDS-page of the main size-exclusion chromatography peak fraction corresponding to $EmbB_2$-$AcpM_2$ complex imaged by Coomassie Brilliant Blue (left panel) and silver stain (right panel). WT protein on the left lane was purified following the cryo-EM sample preparation protocol while the one on the middle lane following the enzymatic activity purification protocol. (**D**) Peptide mass fingerprint analysis of AcpM. Identified peptides are colored in sequence. (**E**) Further identification of EmbB $\alpha(1\rightarrow3)$ arabinosyltransferase activity. The 3-OH position of the terminal arabinose of NV6 was replaced by an azide group (NV13), which could not be turned over by EmbB; but could be by purified EmbC that catalyzes the formation of an $\alpha(1\rightarrow5)$ linkage and AftB from a source of *Msm* membranes that catalyzes the formation of a $\beta(1\rightarrow2)$ linkage. The control AftB *Ms*-membrane activity reported in each independent TLC-autoradiogram (left and right-hand panels) results from the same original assay sample split into two and was included on both plates as a reference point for AftB-activity and NV14 for the EmbC (left-hand panel) and EmbB (right-hand panel). **(F)** 2D-HSQC NMR spectra of purified cell wall AG from (up left) wild type *M. smegmatis* (*Ms*-WT), (up right) *M. smegmatis* Δ*embB* (*Ms*Δ6389) and (down left) *Ms*Δ6389 complemented with a triple-alanine mutant (R249, R253, and R454). The *embB* knockout lacks the 3-arm branching at the terminus of AG. **(G)** Mass spectrometry analysis of solvent extracted DPA from purified *Msm* EmbB.

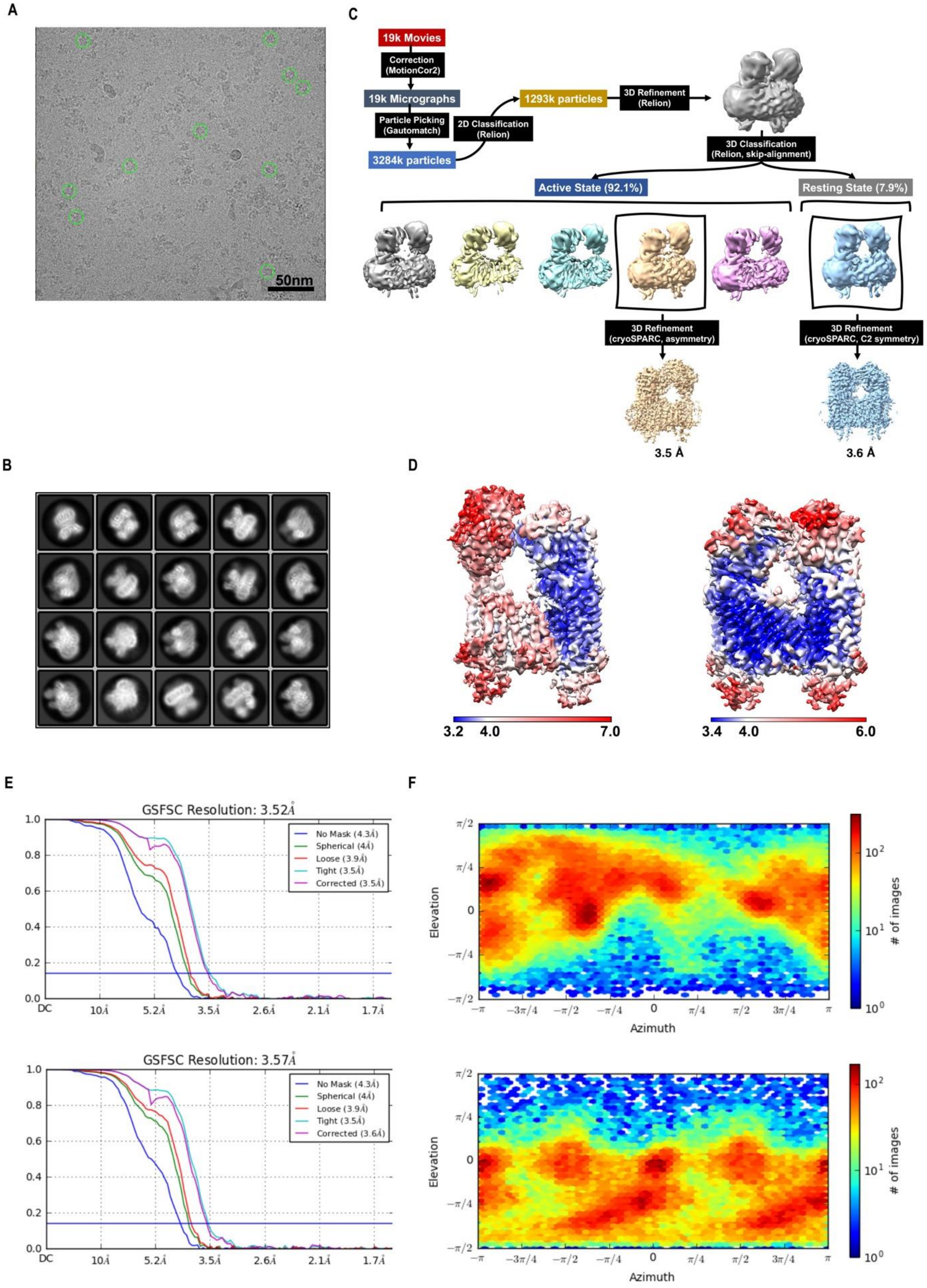
A
50nm
C
19k Movies
Correction (MotionCor2)
19k Micrographs
Particle Picking (Gautomatch)
3284k particles
2D Classification (Relion)
1293k particles
3D Refinement (Relion)
3D Classification (Relion, skip-alignment)
Active State (92.1%)
Resting State (7.9%)
3D Refinement (cryoSPARC, asymmetry)
3D Refinement (cryoSPARC, C2 symmetry)
3.5 Å
3.6 Å
B
D
3.2 4.0 7.0
3.4 4.0 6.0
E
GSFSC Resolution: 3.52Å
No Mask (4.3Å)
Spherical (4Å)
Loose (3.9Å)
Tight (3.5Å)
Corrected (3.5Å)
GSFSC Resolution: 3.57Å
No Mask (4.3Å)
Spherical (4Å)
Loose (3.9Å)
Tight (3.5Å)
Corrected (3.6Å)
DC 10Å 5.2Å 3.5Å 2.6Å 2.1Å 1.7Å
F
Elevation
Azimuth
# of images

Figure S2. Cryo-EM data processing, related to Figure 2.

(**A**) Cryo-EM micrograph of EmbB$_2$-AcpM$_2$. (**B**) 2D class averages of representative orientations observed during processing. (**C**) Flow chart of the processing of cryo-EM data and the discovery of EmbB in two different conformations. (**D**) Local resolution maps calculated by crysoSPARC, sharpened with B-factor values from cryoSPARC (left: “DPA-bound” active state, right: “resting” state). (**E**) FSC of “DPA-bound” active state (upper panel) from the cryoSPARC reconstruction and FSC of “resting” state (lower panel) from the cryoSPARC reconstruction with C2-symmetry applied. (**F**) Orientation distributions in the final reconstruction from cryoSPARC (upper panel: “DPA-bound” active state, lower panel: “resting” state).

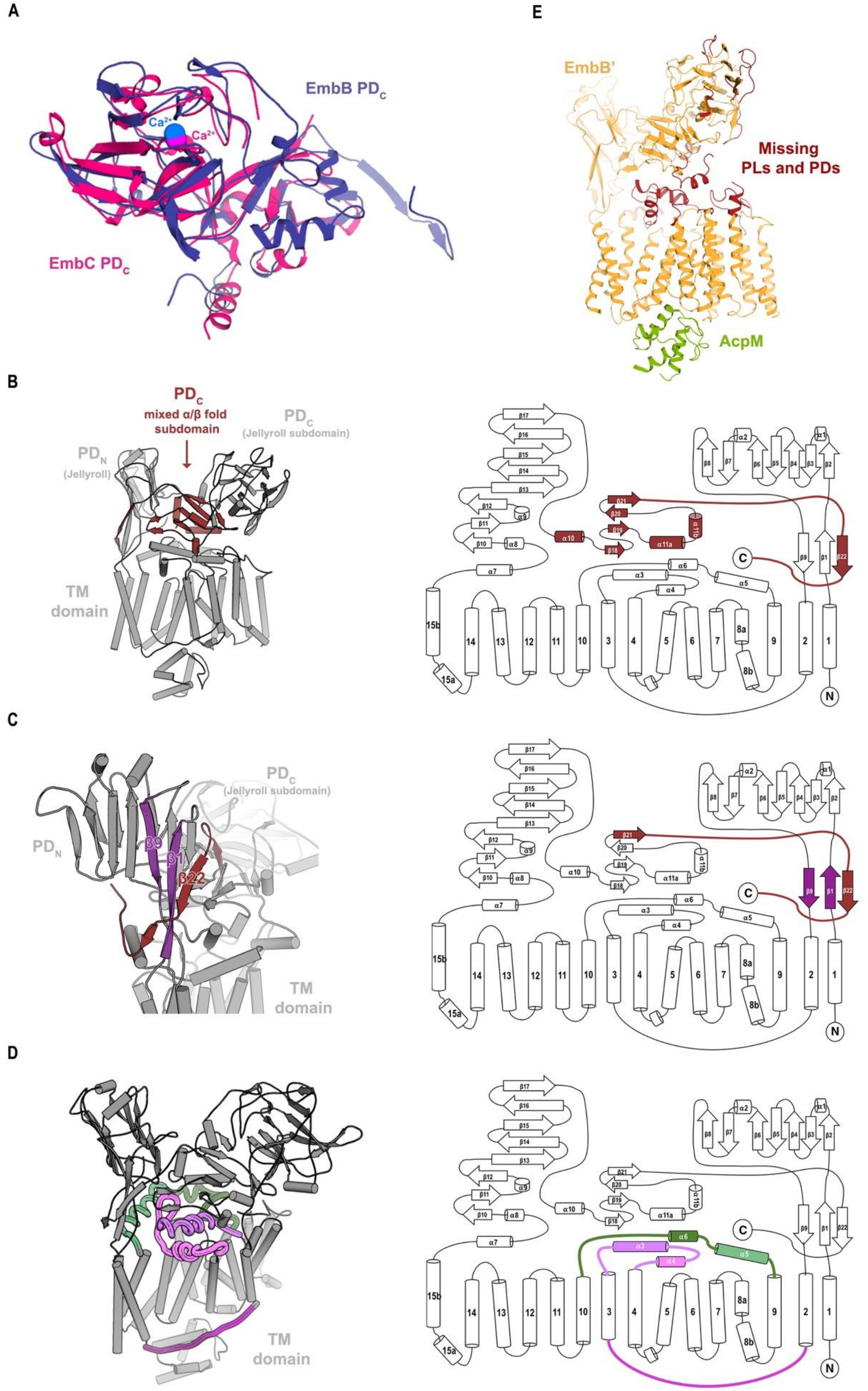
A
EmbB PD$_C$
Ca^{2+}
Ca^{2+}
EmbC PD$_C$
E
EmbB'
Missing PLs and PDs
AcpM
B
PD$_C$ mixed α/β fold subdomain
PD$_C$ (Jellyroll subdomain)
PD$_N$ (Jellyroll)
TM domain
C
PD$_C$ (Jellyroll subdomain)
PD$_N$
β9
β1
β22
TM domain
D
TM domain
C
N

Figure S3. Structural domains of EmbB, related to Figures 2 and 5.

(**A)** PD_C domain of the *Msm*-EmbB protomer superposed on to the C-terminal soluble domain of *Mtb*-EmbC (Alderwick et al., 2011). (**B**) The α/β mixed subdomain in PD_C of EmbB and its relation to the other domains. (**C**) Three-stranded β-sheet formed by PD_N and the tail of PD_C that stabilizes the periplasmic domain. (**D**) CL1 (interacting with AcpM) and PL5 (harboring catalytic motif) of DPA-bound EmbB protomer. **(E)** The missing PLs and PDs (red) of the unbound protomer (yellow) of the DPA-bound state.

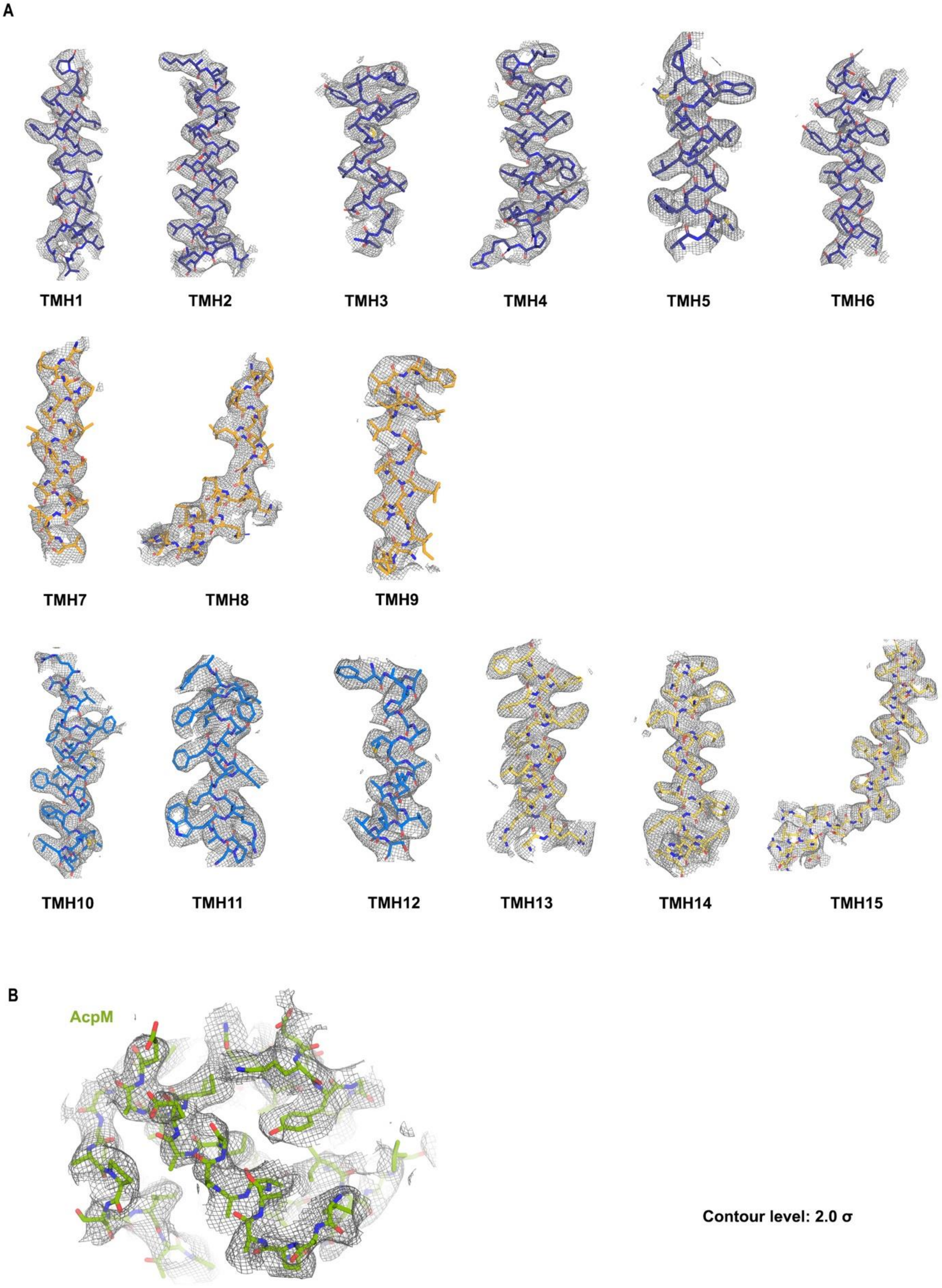
A
TMH1
TMH2
TMH3
TMH4
TMH5
TMH6
TMH7
TMH8
TMH9
TMH10
TMH11
TMH12
TMH13
TMH14
TMH15
B
AcpM
Contour level: 2.0 σ

Figure S4. Representative regions of cryo-EM maps, related to Figure 2.

Fit of the all fifteen TM helices from the protomers in the two states of the $EmbB_2$-$AcpM_2$ complex. (**A)** TMH1-6 from the “DPA-bound” protomer of the active state. TMH7-9 from the “DPA-unbound protomer” of the active state. TMH10-12 and TMH13-15 from two protomers of “resting” state. (**B**) AcpM bound to the DPA-bound protomer in the active state.

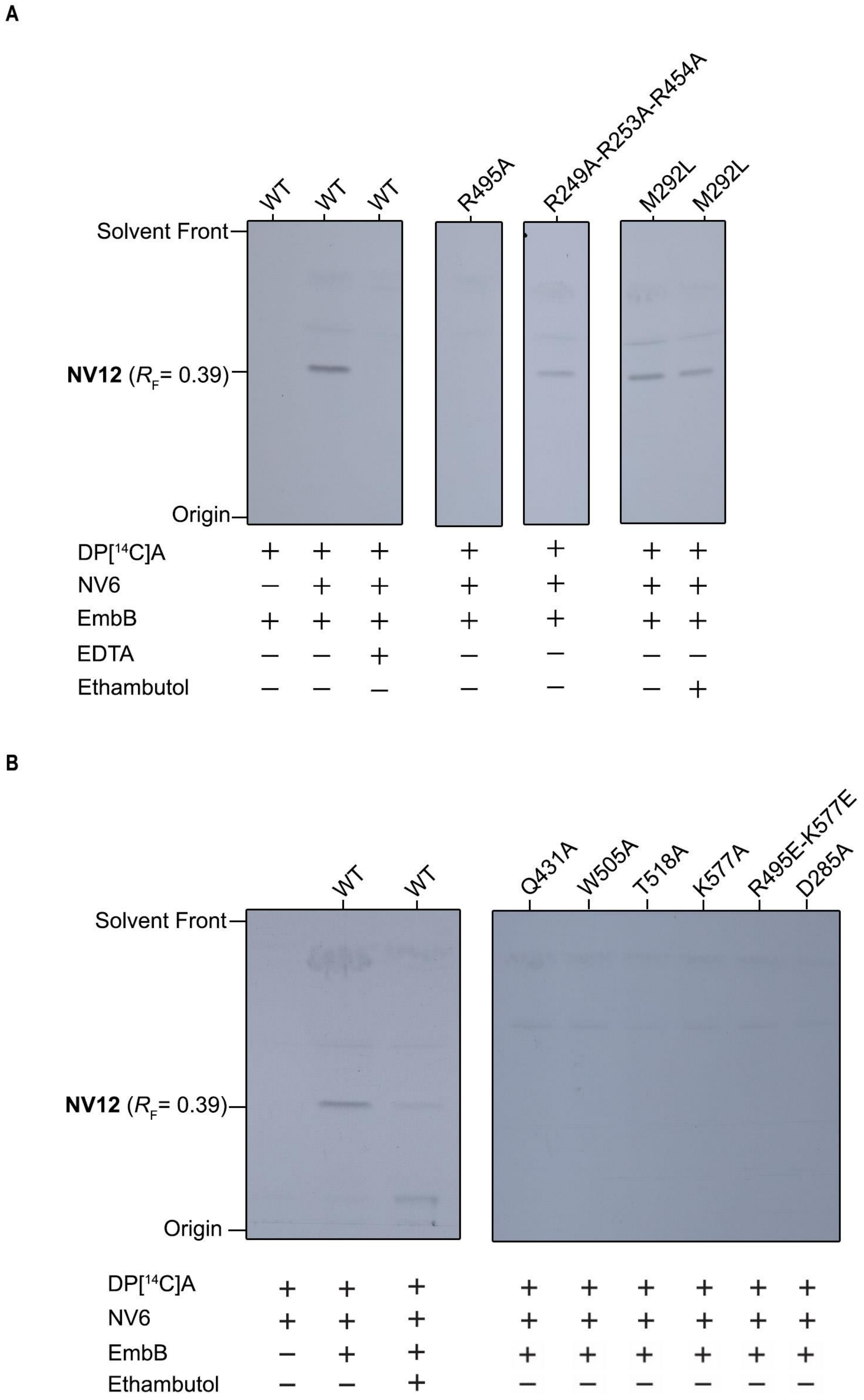
A
WT
WT
WT
R495A
R249A-R253A-R454A
M292L
M292L
Solvent Front
NV12 (R_F= 0.39)
Origin
DP[14C]A + + + + + + +
NV6 − + + + + + +
EmbB + + + + + + +
EDTA − − + − − − −
Ethambutol − − − − − − +
B
WT
WT
Q431A
W505A
T518A
K577A
R495E-K577E
D285A
Solvent Front
NV12 (R_F= 0.39)
Origin
DP[14C]A + + + + + + + + +
NV6 + + + + + + + + +
EmbB − + + + + + + + +
Ethambutol − − + − − − − − −

Figure S5. EmbB and related mutated proteins in a functional arabinosyltransferase assay, related to Figures 3 and 4.

(**A)** and (**B)** represent two individual experiments. To be noted, the left three control lanes in panel (B) are also referenced in Figure 1B as they came from the same experiment.

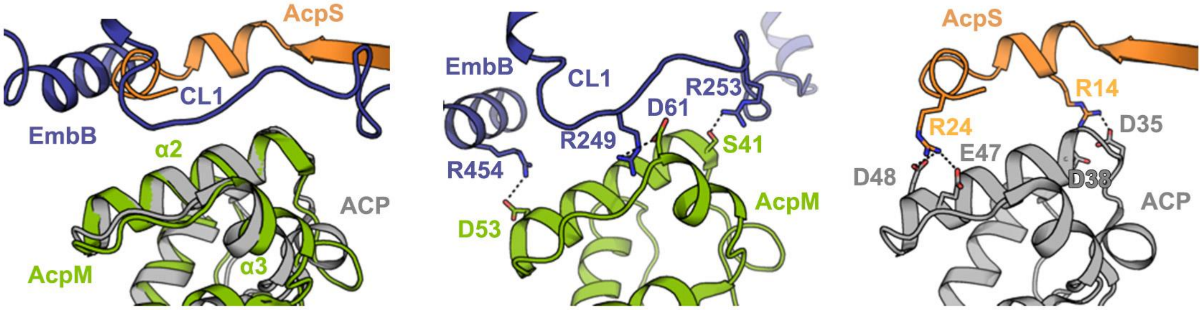

Figure S6. Comparison of the EmbB-AcpM binding interface with the AcpS-ACP binding interface, related to Figure 4.

(left) Superimposition of the AcpM in complex with EmbB (blue) and AcpS (orange) , and specific interactions of AcpM in complex with EmbB(middle) and with AcpS (right).

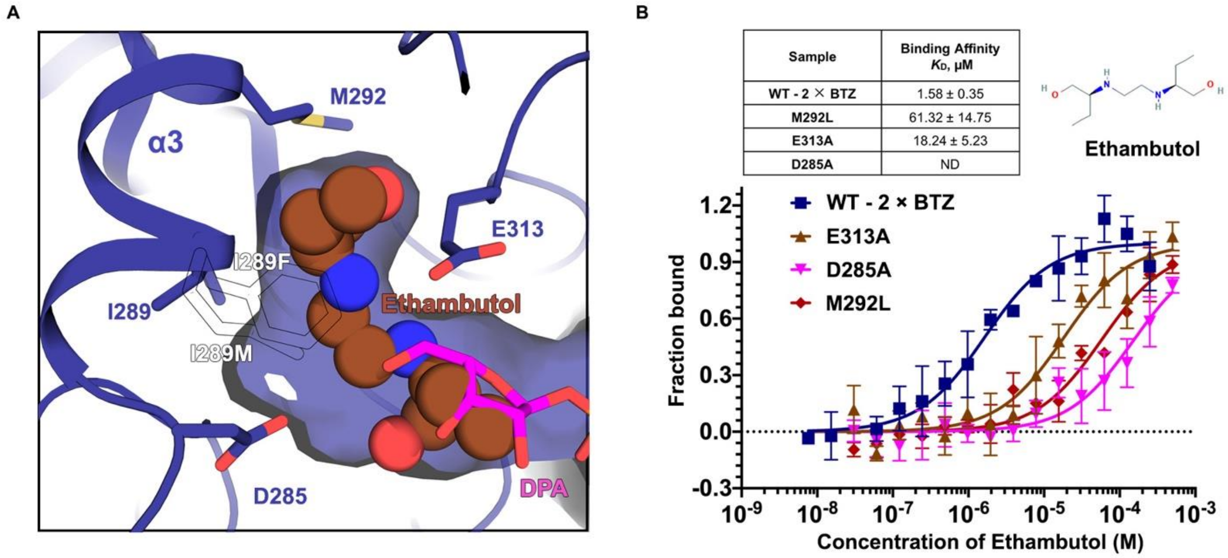

Figure S7. Probing the possible binding location of ethambutol, related to Figure 7. **(A)** Molecular docking of ethambutol (shown as sphere) in the DPA (in magenta) binding pocket (shown as surface). I289M and I289F are two mutations leading to ethambutol resistance in an *Msm* study. They are indicated by black-outlined side chains. It is likely that these changes can sterically hinder ethambutol binding. **(B)** MST assay results for the binding ethambutol to the EmbB mutants E313A and D285A. To be noted, the results of wild-type EmbB treated with 2 × MIC of BTZ-043 and mutant M292L in Figure 7B are also shown here as controls and references. They came from the same experiment with the mutants E313A and D285A. ND, K_D could not be determined.

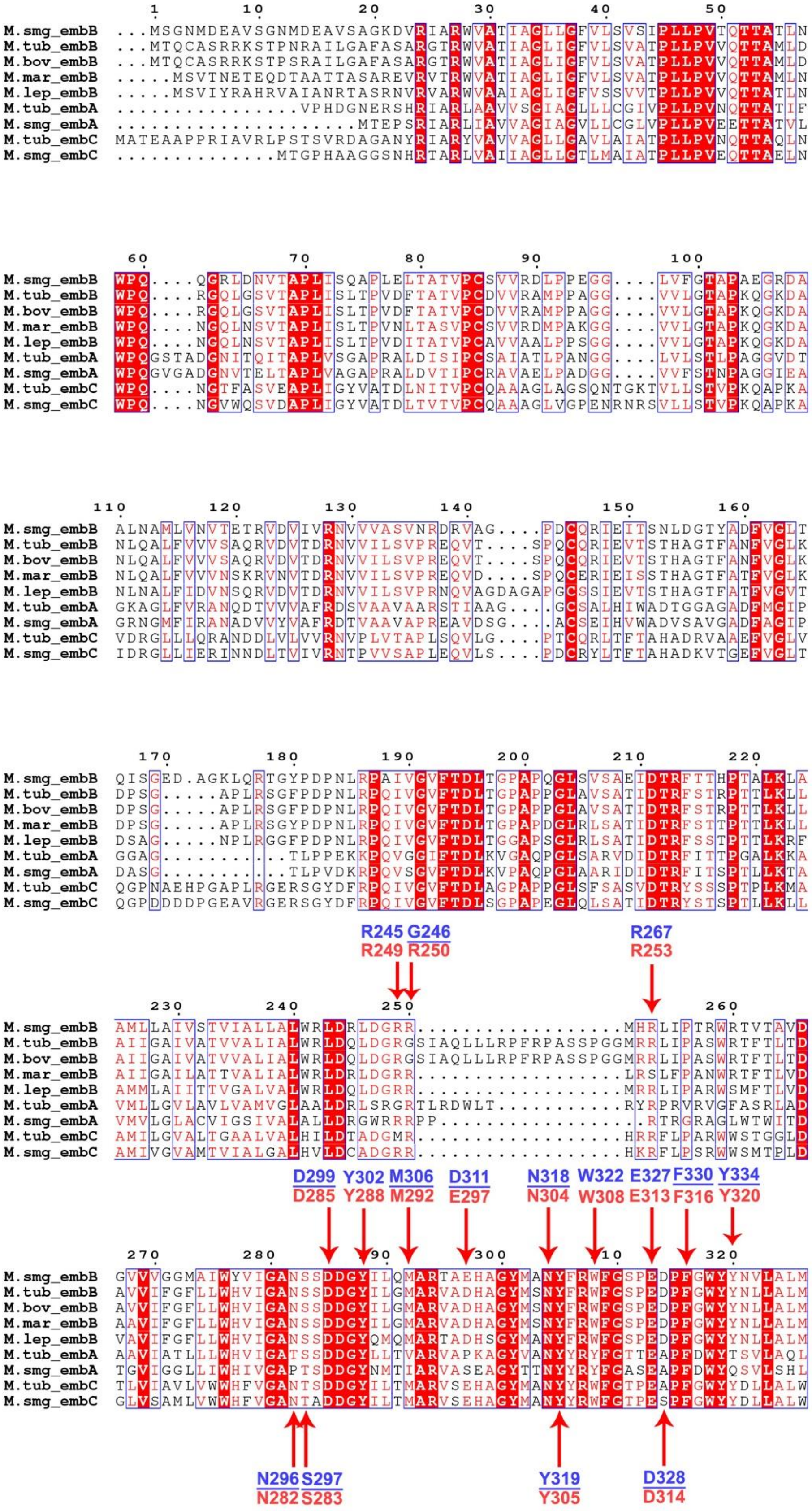
M.smg_embB
M.tub_embB
M.bov_embB
M.mar_embB
M.lep_embB
M.tub_embA
M.smg_embA
M.tub_embC
M.smg_embC
R245 G246
R249 R250
R267
R253
D299 Y302 M306 D311 N318 W322 E327 F330 Y334
D285 Y288 M292 E297 N304 W308 E313 F316 Y320
N296 S297
N282 S283
Y319
Y305
D328
D314

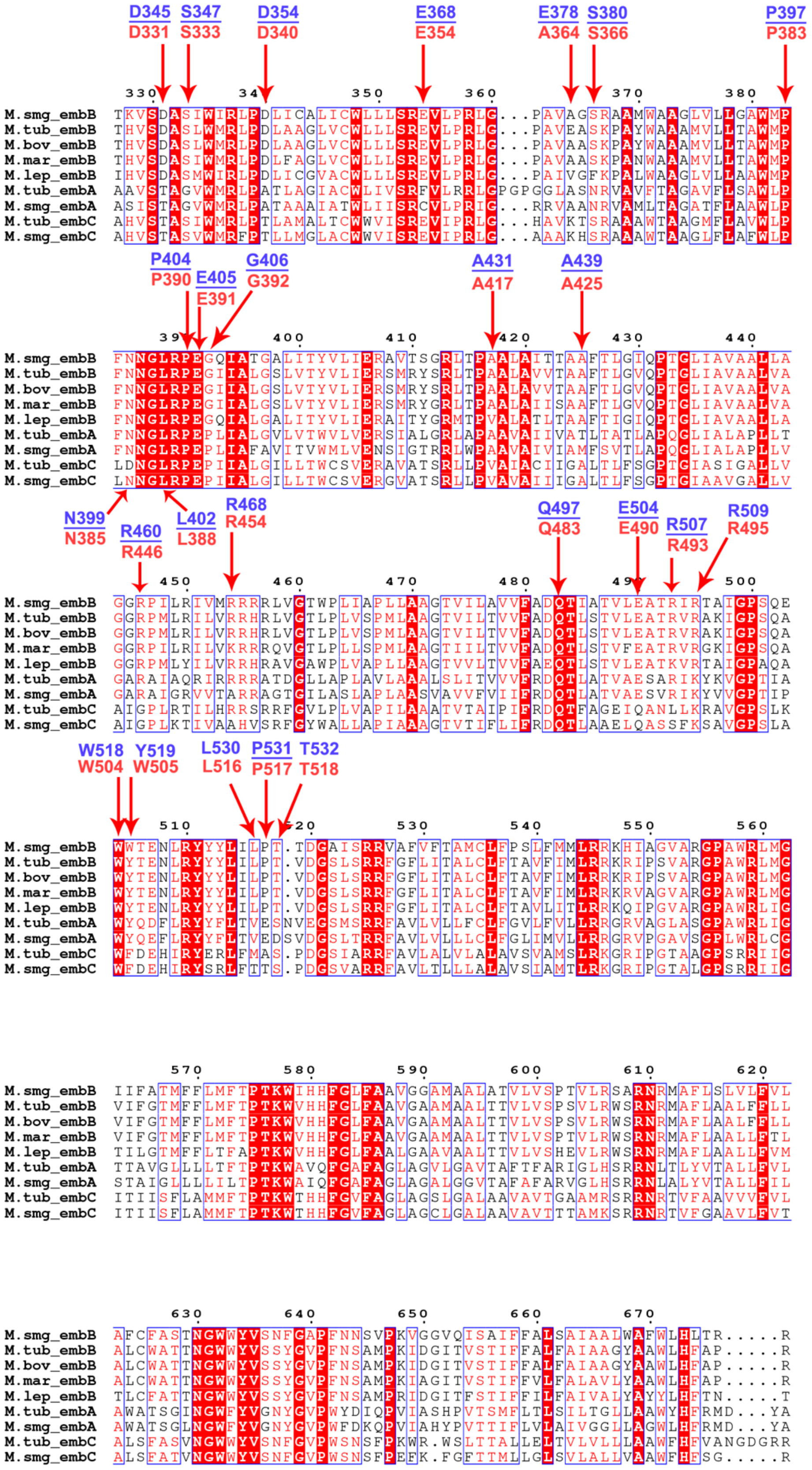

D345 D331
S347 S333
D354 D340
E368 E354
E378 A364
S380 S366
P397 P383
330 340 350 360 370 380
M.smg_embB TKVSDASIWIRLPDLICALICWLLLSREVLPRLG...PAVAGSRAAMWAAGLVLLGAWMP
M.tub_embB THVSDASLWMRLPDLAAGLVCWLLLSREVLPRLG...PAVEASKPAYWAAAMVLLTAWMP
M.bov_embB THVSDASLWMRLPDLAAGLVCWLLLSREVLPRLG...PAVAASKPAYWAAAMVLLTAWMP
M.mar_embB THVSDASIWMRLPDLFAGLVCWLLLSREVLPRLG...PAVAASKPANWAAAMVLLTAWMP
M.lep_embB IHVSDASMWIRLPDLICGVACWLLLSREVLPRLG...PAIVGFKPALWAAGLVLLAAWMP
M.tub_embA AAVSTAGVWMRLPATLAGIACWLIVSRFVLRRLGPGPGGLASNRVAVFTAGAVFLSAWLP
M.smg_embA ASISTAGVWMRLPATAAIATWLIISRCVLPRIG...RRVAANRVAMLTAGATFLAAWLP
M.tub_embC AHVSTASIWMRLPTLAMALTCWWVISREVIPRLG...HAVKTSRAAAWTAAGMFLAVWLP
M.smg_embC AHVSTASVWMRFPTLLMGLACWWVISREVIPRLG...AAAKHSRAAAWTAAGLFLAFWLP
P404 P390
E405 E391
G406 G392
A431 A417
A439 A425
390 400 410 420 430 440
M.smg_embB FNNGLRPEGQIATGALITYVLIERAVTSGRLTPAALAITTAAFTLGIQPTGLIAVAALLA
M.tub_embB FNNGLRPEGIIALGSLVTYVLIERSMRYSRLTPAALAVVTAAFTLGVQPTGLIAVAALVA
M.bov_embB FNNGLRPEGIIALGSLVTYVLIERSMRYSRLTPAALAVVTAAFTLGVQPTGLIAVAALVA
M.mar_embB FNNGLRPEGIIALGSLVTYVLIERSMRYGRLTPAALAIISAAFTLGVQPTGLIAVAALVA
M.lep_embB FNNGLRPEGQIALGALITYVLIERAITYGRMTPVALATLTAAFTIGIQPTGLIAVAALLA
M.tub_embA FNNGLRPEPLIALGVLVTWVLVERSIALGRLAPAAVAIIVATLTATLAPQGLIALAPLLT
M.smg_embA FNNGLRPEPLIAFAVITVWMLVENSIGTRRLWPAAVAIVIAMFSVTLAPQGLIALAPLLV
M.tub_embC LDNGLRPEPIIALGILLTWCSVERAVATSRLLPVAIACIIGALTLFSGPTGIASIGALLV
M.smg_embC LNNGLRPEPIIALGILLTWCSVERGVATSRLLPVAVAIIIGALTLFSGPTGIAAVGALLV
N399 N385
L402 L388
R460 R446
R468 R454
Q497 Q483
E504 E490
R507 R493
R509 R495
450 460 470 480 490 500
M.smg_embB GGRPILRIVMRRRRLVGTWPLIAPLLAAGTVILAVVFADQTIATVLEATRIRTAIGPSQE
M.tub_embB GGRPMLRILVRRHRLVGTLPLVSPMLAAGTVILTVVFADQTLSTVLEATRVRAKIGPSQA
M.bov_embB GGRPMLRILVRRHRLVGTLPLVSPMLAAGTVILTVVFADQTLSTVLEATRVRAKIGPSQA
M.mar_embB GGRPILRILVKRRRQVGTLPLLSPMLAAGTIILTVVFADQTLSTVFEATRVRGKIGPSQA
M.lep_embB GGRPMLYILVRRHRAVGAWPLVAPLLAAGTVVLTVVFAEQTLSTVLEATKVRTAIGPAQA
M.tub_embA GARAIAQRIRRRRATDGLLAPLAVLAAALSLITVVFRDQTLATVAESARIKYKVGPTIA
M.smg_embA GARAIGRVVTARRAGTGILASLAPLAASVAVVFVIIFRDQTLATVAESVRIKYVVGPTIP
M.tub_embC AIGPLRTILHRRSRRFGVLPLVAPILAAATVTAIPIFRDQTFAGEIQANLLKRAVGPSLK
M.smg_embC AIGPLKTIVAAHVSRFGYWALLAPIAAAGTVTIFLIFRDQTLAAELQASSFKSAVGPSLA
W518 W504
Y519 W505
L530 L516
P531 P517
T532 T518
510 520 530 540 550 560
M.smg_embB WWTENLRYYYLILPT.TDGAISRRVAFVFTAMCLFPSLFMMLRRKHIAGVARGPAWRLMG
M.tub_embB WYTENLRYYYLILPT.VDGSLSRRFGFLITALCLFTAVFIMLRRKRIPSVARGPAWRLMG
M.bov_embB WYTENLRYYYLILPT.VDGSLSRRFGFLITALCLFTAVFIMLRRKRIPSVARGPAWRLMG
M.mar_embB WYTENLRYYYLILPT.VDGSLSRRFGFLITALCLFTAVFIMLRRKRVAGVARGPAWRLMG
M.lep_embB WYTENLRYYYLILPT.VDGSLSRRFGFLITALCLFTAVLITLRRKQIPGVARGPAWRLIG
M.tub_embA WYQDFLRYYFLTVESNVEGSMSRRFAVLVLLFCLFGVLFVLLRRGRVAGLASGPAWRLIG
M.smg_embA WYQEFLRYYFLTVEDSVDGSLTRRFAVLVLLCLFGLIMVLLRRGRVPGAVSGPLWRLCG
M.tub_embC WFDEHIRYERLFMAS.PDGSIARRFAVLALVLALAVSVAMSLRKGRIPGTAAGPSRRIIG
M.smg_embC WFDEHIRYSRLFTTS.PDGSVARRFAVLTLLLALAVSIAMTLRKGRIPGTALGPSRRIIG
570 580 590 600 610 620
M.smg_embB IIFATMFFLMFTPTKWIHHFGLFAAVGGAMAALATVLVSPTVLRSARNRMAFLSLVLFVL
M.tub_embB VIFGTMFFLMFTPTKWVHHFGLFAAVGAAMAALTTVLVSPSVLRWSRNRMAFLAALFFLL
M.bov_embB VIFGTMFFLMFTPTKWVHHFGLFAAVGAAMAALTTVLVSPSVLRWSRNRMAFLAALFFLL
M.mar_embB VIFGTMFFLMFTPTKWVHHFGLFAAVGAAMAALTTVLVSPTVLRWSRNRMAFLAALLFTL
M.lep_embB TILGTMFFLTFAPTKWVHHFGLFAALGAAVAALTTVLVSHEVLRWSRNRMAFLAALLFVM
M.tub_embA TTAVGLLLLTFTPTKWAVQFGAFAGLAGVLGAVTAFTFARIGLHSRRNLTLYVTALLFVL
M.smg_embA STAIGLLLLILTPTKWAIQFGAFAGLAGALGGVTAFAFARVGLHSRRNLALYVTALLFIL
M.tub_embC ITIISFLAMMFTPTKWTHHFGVFAGLAGSLGALAAVAVTGAAMRSRRNRTVFAAVVVFVL
M.smg_embC ITIISFLAMMFTPTKWTHHFGVFAGLAGCLGALAAVAVTTTAMKSRRNRTVFGAAVLFVT
630 640 650 660 670
M.smg_embB AFCFASTNGWWYVSNFGAPFNNSVPKVGGVQISAIFFALSAIAALWAFWLHLTR.....R
M.tub_embB ALCWATTNGWWYVSSYGVPFNSAMPKIDGITVSTIFFALFAIAAGYAAWLHFAP.....R
M.bov_embB ALCWATTNGWWYVSSYGVPFNSAMPKIDGITVSTIFFALFAIAAGYAAWLHFAP.....R
M.mar_embB ALCWATTNGWWYVSSYGVPFNSAMPKIAGITVSTIFFVLFALAVLYAAWLHFAP.....R
M.lep_embB TLCFATTNGWWYVSSYGVPFNSAMPRIDGITFSTIFFILFAIVALYAYYLHFTN.....T
M.tub_embA AWATSGINGWFYVGNYGVPWYDIQPVIASHPVTSMFLTLLSILTGLLAAWYHFRMD...YA
M.smg_embA AWATSGLNGWFYVGNYGVPWFDKQPVIAHYPVTTIFLVLAIVGGLLAGWLHFRMD...YA
M.tub_embC ALSFASVNGWWYVSNFGVPWSNSFPKWR.WSLTTALLELTVLVLLAAWFHFVANGDGRR
M.smg_embC ALSFATVNGWWYVSNFGVPWSNSFPEFK.FGFTTMLGLSVLALLVAAWFHFSG.....R

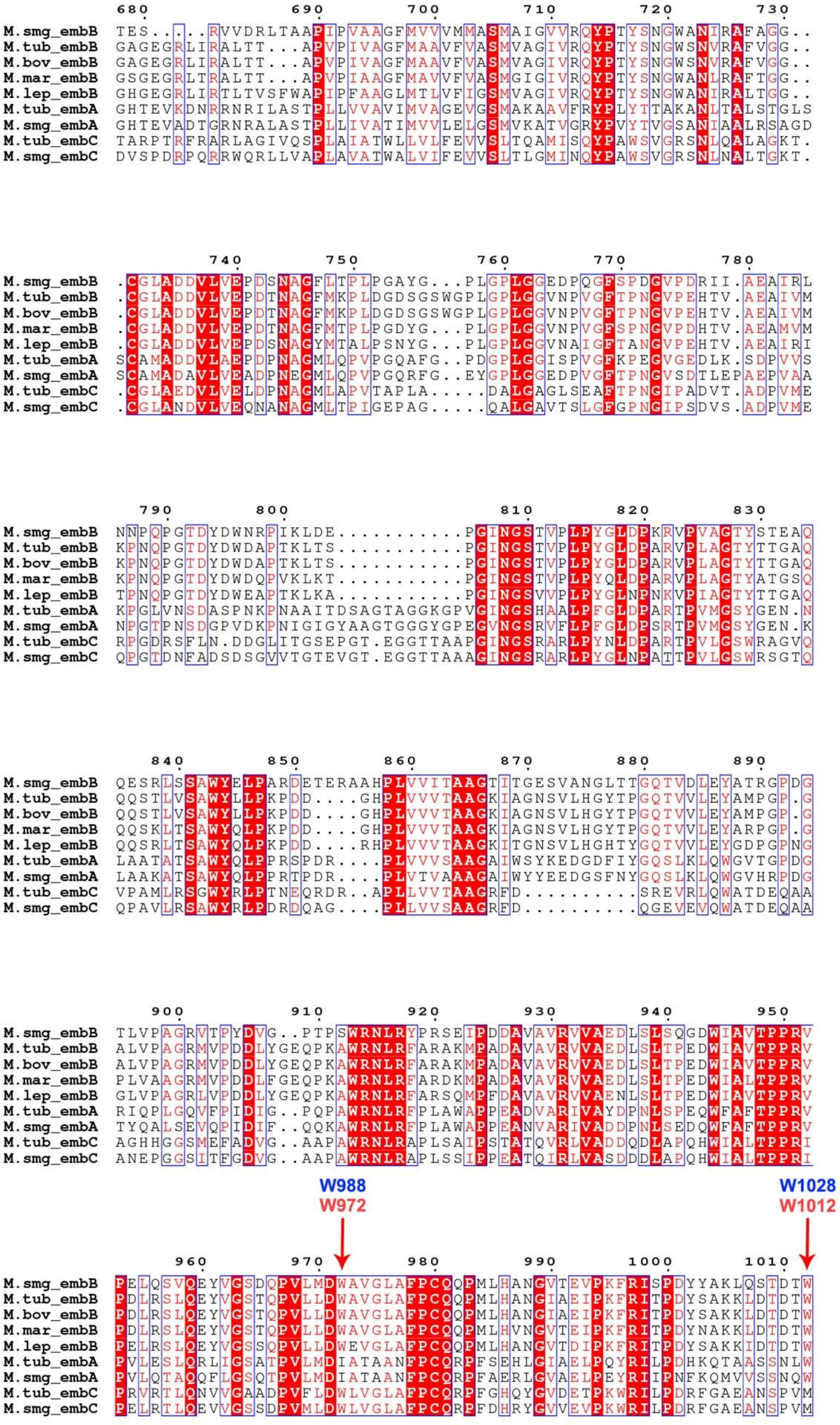
W988
W972
W1028
W1012
M.smg_embB
M.tub_embB
M.bov_embB
M.mar_embB
M.lep_embB
M.tub_embA
M.smg_embA
M.tub_embC
M.smg_embC

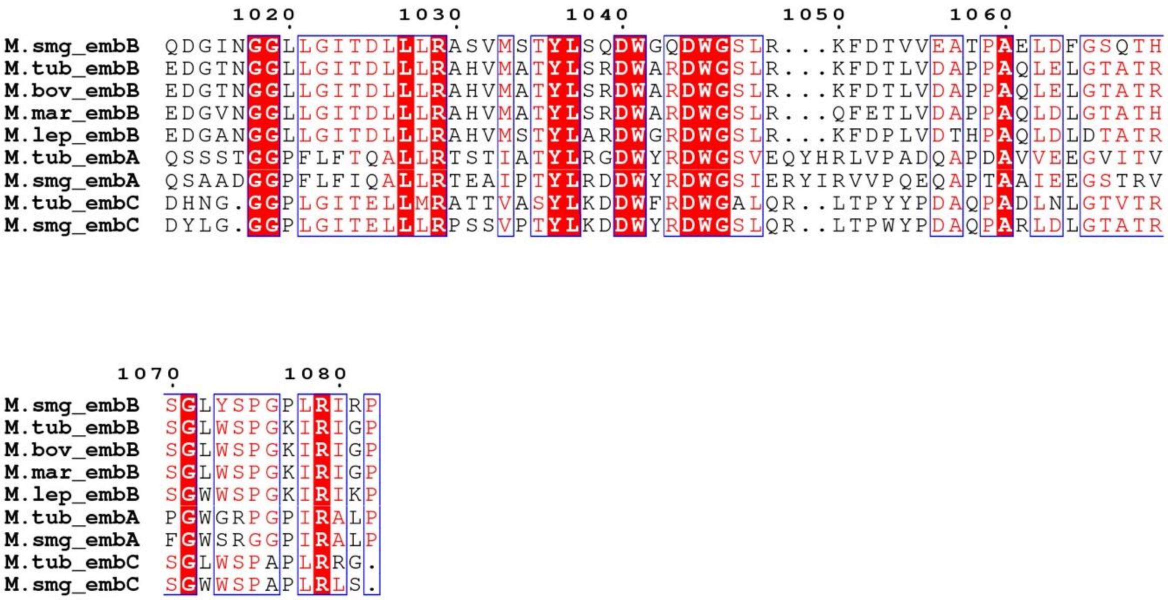

Figure S8. Sequence alignment of EmbB in five representative mycobacterial species and alignment with EmbA/C in *Mtb/Msm*, related to Figures 3 and 7.

Highly or partially conserved residues of functional importance or ethambutol resistant associated sites are identified with red arrows. All amino acids in red are from *Msm* and in blue are their equivalence from *Mtb*, clinical ethambutol resistant mutant sites are underlined.

A

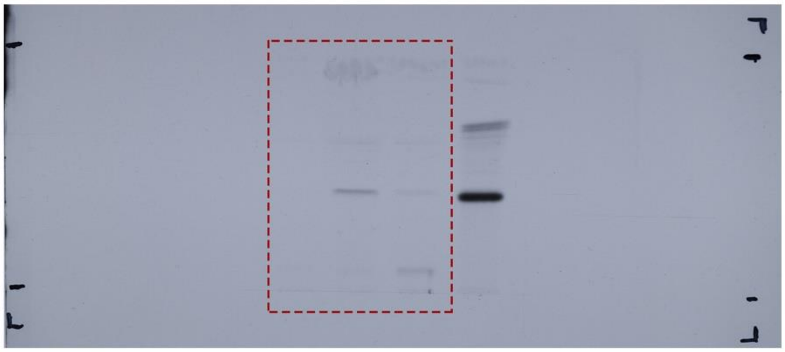

B

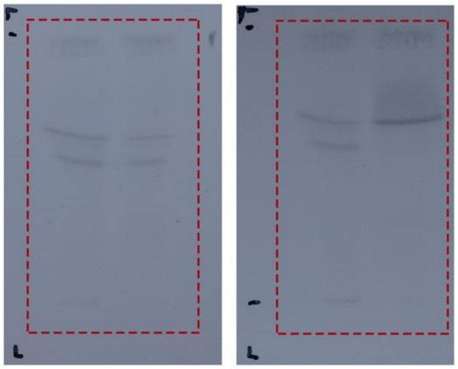

C

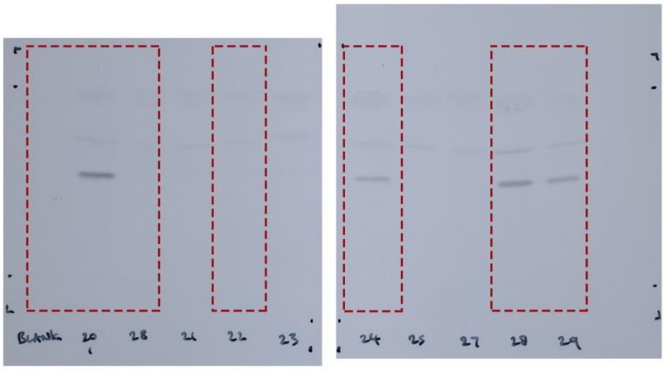

D

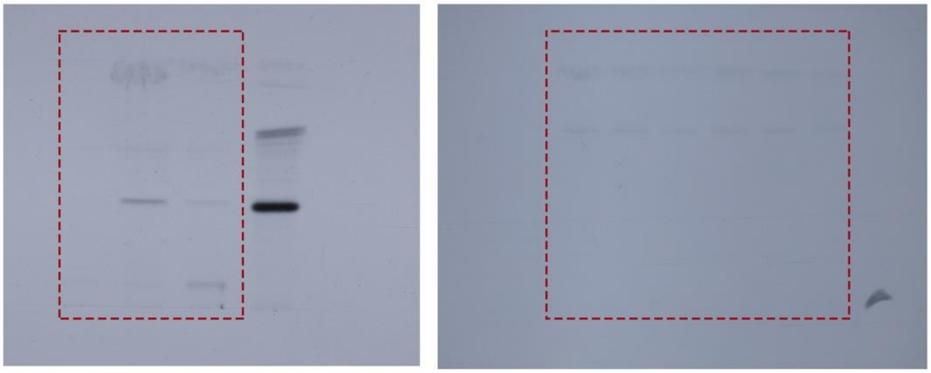

Figure S9. The original TLC plates and how they were used to make the panel of figure 1B (A), figure S1F (B), figure S5A (C) and figure S5B (D), related to Figures 1 and S5.

Red dashed boxes indicate the lanes that were extracted to assemble the panels. The left panel control in (D) was reused in panel (A) and also figure 1B to demonstrate the activity of the purified enzyme.

Table S1. Statistics of data collection, image processing and model building

**Data Collection**		
**EM equipment**	FEI Titan Krios	
**Voltage (kV)**	300	
**Detector**	Gatan K2	
**Pixel Size (Å/pixel)**	0.82	
**Electron dose (e^-/Å^2)**	60	
**Defocus range (μm)**	1.5~2.5	
**Reconstruction**		
**Software**	Relion 2.1 / cryoSPARC	
**Map Name**	Heterodimer	Homodimer
**Number of used particles**	125899	84483
**Symmetry**	C1	C2
**Map sharpening B-factor (Å^2)**	-151	-166
**Final Resolution (Å)**	3.5	3.6
**Model Building**		
**Software**	Coot	
**Model Refinement**		
**Software**	PHENIX	
**Model name**	Heterodimer	Homodimer
**Map CC (whole unit cell)**	0.79	0.78
**Map CC (around atoms)**	0.81	0.80
**RMSD (bonds)(Å)**	0.007	0.008
**RMSD (angles)(º)**	1.271	1.302
**Model Composition**		
**Model name**	Heterodimer	Homodimer
**Protein residues**	2064	2232
**DPA**	1	-
**AcpM**	2	2
**Validation**		
**Ramachandran plot**		
**Model name**	Heterodimer	Homodimer
**Outliers (%)**	0.05	0.09
**Allowed (%)**	14.22	11.55
**Favored (%)**	85.74	88.36
**Rotamer outliers (%)**	1.55	1.37

260

Table S2. Summary of the Model

	Protomer Name	Chain	Total residues /Range built	Poly-ALA model	Un-modelled residues	Rigid Docking	% atomic model	Ligands	Resolution (Å)
**Hetero dimer**	EmbB Protomer 1	A	1082/ 23-168, 170-676, 678-1082	-	1-22, 169, 677	-	97.8%	DPA	3.2-5.0
	EmbB Protomer 2	B	1082/ 23-167, 173-278, 339-489, 525-626, 647-677, 680-705	217-219,382-386,647-649	1-22, 168-172, 279-338, 490-524, 627-646, 678-679, 706-732, 752-760, 831-834, 848-851, 874-879, 890-901, 926-928, 1004-1018, 1075-1082	733-1074	51.8%	-	3.8-7.0
	AcpM	C/D	99/ 3-80	-	1-2, 81-99	-	78.8%	-	3.6-6.8
**Homo dimer**	EmbB Protomer 1/2	A/B	1082/ 23-140, 145-502, 521-1082	-	1-22, 141-144, 503-520	-	95.9%	-	3.4-6.0
	AcpM	C/D	99/ 3-80	-	1-2, 81-99	-	78.8%	-	3.7-6.0

References and Notes:

Adams, P.D., Afonine, P.V., Bunkoczi, G., Chen, V.B., Davis, I.W., Echols, N., Headd, J.J., Hung, L.W., Kapral, G.J., Grosse-Kunstleve, R.W., *et al.* (2010). PHENIX: a comprehensive Python-based system for macromolecular structure solution. Acta Crystallogr D Biol Crystallogr *66*, 213-221.

Alderwick, L.J., Lloyd, G.S., Ghadbane, H., May, J.W., Bhatt, A., Eggeling, L., Futterer, K., and Besra, G.S. (2011). The C-terminal domain of the Arabinosyltransferase Mycobacterium tuberculosis EmbC is a lectin-like carbohydrate binding module. Plos Pathog *7*, e1001299.

Banks, J.L., Beard, H.S., Cao, Y., Cho, A.E., Damm, W., Farid, R., Felts, A.K., Halgren, T.A., Mainz, D.T., Maple, J.R., *et al.* (2005). Integrated Modeling Program, Applied Chemical Theory (IMPACT). J Comput Chem *26*, 1752-1780.

DeLano, W. (2010). The PyMOL molecular graphics system. Schrödinger, LLC, New York.

Emsley, P., Lohkamp, B., Scott, W.G., and Cowtan, K. (2010). Features and development of Coot. Acta Crystallogr D Biol Crystallogr *66*, 486-501.

Friesner, R.A., Banks, J.L., Murphy, R.B., Halgren, T.A., Klicic, J.J., Mainz, D.T., Repasky, M.P., Knoll, E.H., Shelley, M., Perry, J.K., *et al.* (2004). Glide: a new approach for rapid, accurate docking and scoring. 1. Method and assessment of docking accuracy. J Med Chem *47*, 1739-1749.

Goddard, T.D., Huang, C.C., Meng, E.C., Pettersen, E.F., Couch, G.S., Morris, J.H., and Ferrin, T.E. (2018). UCSF ChimeraX: Meeting modern challenges in visualization and analysis. Protein Sci *27*, 14-25.

Kelley, L.A., Mezulis, S., Yates, C.M., Wass, M.N., and Sternberg, M.J. (2015). The Phyre2 web portal for protein modeling, prediction and analysis. Nat Protoc *10*, 845-858.

Kimanius, D., Forsberg, B.O., Scheres, S.H.W., and Lindahl, E. (2016). Accelerated cryo-EM structure determination with parallelisation using GPUs in RELION-2. Elife *5*, e18722.

Lee, R.E., Brennan, P.J., and Besra, G.S. (1997). Mycobacterial arabinan biosynthesis: the use of synthetic arabinoside acceptors in the development of an arabinosyl transfer assay. Glycobiology *7*, 1121-1128.

Mastronarde, D.N. (2003). SerialEM: A Program for Automated Tilt Series Acquisition on Tecnai Microscopes Using Prediction of Specimen Position. Microscopy and Microanalysis *9*, 1182-1183.

Murshudov, G.N., Skubak, P., Lebedev, A.A., Pannu, N.S., Steiner, R.A., Nicholls, R.A., Winn, M.D., Long, F., and Vagin, A.A. (2011). REFMAC5 for the refinement of macromolecular crystal structures. Acta Crystallogr D Biol Crystallogr *67*, 355-367.

Pettersen, E.F., Goddard, T.D., Huang, C.C., Couch, G.S., Greenblatt, D.M., Meng, E.C., and Ferrin, T.E. (2004). UCSF Chimera--a visualization system for exploratory research and analysis. J Comput Chem *25*, 1605-1612.

Punjani, A., Rubinstein, J.L., Fleet, D.J., and Brubaker, M.A. (2017). cryoSPARC: algorithms for rapid unsupervised cryo-EM structure determination. Nat Methods *14*, 290-296.

Ramirez-Aportela, E., Vilas, J.L., Melero, R., Conesa, P., Martinez, M., Maluenda, D., Mota, J., Jimenez, A., Vargas, J., and Marabini, R. (2018). Automatic local resolution-based sharpening of cryo-EM maps. bioRxiv, 433284.

Rosenthal, P.B., and Henderson, R. (2003). Optimal determination of particle orientation, absolute hand, and contrast loss in single-particle electron cryomicroscopy. J Mol Biol *333*, 721-745.

Scheres, S.H. (2012a). A Bayesian view on cryo-EM structure determination. J Mol Biol *415*, 406-418.

Scheres, S.H. (2012b). RELION: implementation of a Bayesian approach to cryo-EM structure determination. J Struct Biol *180*, 519-530.

Zhang, B., Li, J., Yang, X., Wu, L., Zhang, J., Yang, Y., Zhao, Y., Zhang, L., Yang, X., Yang, X., *et al.* (2019). Crystal Structures of Membrane Transporter MmpL3, an Anti-TB Drug Target. Cell *176*, 636-648 e613.

Zhang, K. (2016). Gctf: Real-time CTF determination and correction. J Struct Biol *193*, 1-12.

Zheng, S.Q., Palovcak, E., Armache, J.P., Verba, K.A., Cheng, Y., and Agard, D.A. (2017). MotionCor2: anisotropic correction of beam-induced motion for improved cryo-electron microscopy. Nat Methods *14*, 331-332.
